# An infection-induced RhoB-Beclin 1-Hsp90 complex enhances clearance of uropathogenic *Escherichia coli*

**DOI:** 10.1038/s41467-021-22726-8

**Published:** 2021-05-10

**Authors:** Chunhui Miao, Mingyu Yu, Geng Pei, Zhenyi Ma, Lisong Zhang, Jianming Yang, Junqiang Lv, Zhi-Song Zhang, Evan T. Keller, Zhi Yao, Quan Wang

**Affiliations:** 1 Department of Immunology, Key Laboratory of Immune Microenvironment and Disease of the Educational Ministry of China, Tianjin Key Laboratory of Cellular and Molecular Immunology, School of Basic Medical Sciences, Tianjin Medical University, Tianjin, China; 2 Department of Biochemistry and Molecular Biology, School of Basic Medical Sciences, Tianjin Key Laboratory of Medical Epigenetics, Tianjin Medical University, Tianjin, China; 3 State Key Laboratory of Medicinal Chemical Biology and College of Pharmacy, Collaborative Innovation Center for Biotherapy, Tianjin Key Laboratory of Molecular Drug Research, Nankai University, Tianjin, China; 4 Department of Urology, University of Michigan, Ann Arbor, MI USA; 5 2011 Collaborative Innovation Center of Tianjin for Medical Epigenetics, Tianjin Medical University, Tianjin, China

**Keywords:** Antimicrobial responses, Pathogens, Urinary tract infection

## Abstract

Host cells use several anti-bacterial pathways to defend against pathogens. Here, using a uropathogenic *Escherichia coli* (UPEC) infection model, we demonstrate that bacterial infection upregulates RhoB, which subsequently promotes intracellular bacteria clearance by inducing LC3 lipidation and autophagosome formation. RhoB binds with Beclin 1 through its residues at 118 to 140 and the Beclin 1 CCD domain, with RhoB Arg133 being the key binding residue. Binding of RhoB to Beclin 1 enhances the Hsp90-Beclin 1 interaction, preventing Beclin 1 degradation. RhoB also directly interacts with Hsp90, maintaining RhoB levels. UPEC infections increase RhoB, Beclin 1 and LC3 levels in bladder epithelium in vivo, whereas Beclin 1 and LC3 levels as well as UPEC clearance are substantially reduced in *RhoB*^+/−^ and *RhoB*^−/−^ mice upon infection. We conclude that when stimulated by UPEC infections, host cells promote UPEC clearance through the RhoB-Beclin 1-HSP90 complex, indicating RhoB may be a useful target when developing UPEC treatment strategies.

## Abstract (web summary)

Bacterial invasion can lead to multiple host cell responses. Here, the authors show that in a model of uropathogenic E. coli, RhoB is upregulated and induces autophagosome formation in a complex with Beclin1 and Hsp90, promoting bacterial clearance.

## Introduction

Many anti-bacterial pathways are involved in host defenses against pathogens. Autophagy serves as a process induced by invading pathogens^1^, and selectively targets invasive bacteria^2^, such as *Mycobacterium tuberculosi*s, *Shigella flexneri, Group A streptococcus,* and *Salmonella*^3–7^, for autolysosome-dependent degradation. However, the detailed mechanisms involved in autophagy-mediated bacterial clearance are diverse.

Uropathogenic *Escherichia coli* (UPEC), a Gram-negative intracellular pathogenic bacterium, is the leading cause of urinary tract infections (UTIs), which have a high incidence and frequent recurrence rate in women^8^. UPEC is reported to reside in autophagosomes during infection^9^. However, ATG16L1 (an autophagy protein) deficient mice lead to rapid clearance of UPEC, through inducing architectural alterations in superficial urothelial cells and enhancing IL-1β-mediated hyperinflammatory response in macrophages^10–13^. On the other hand, significantly increased UPEC bacterial loads are observed in mice with ATG3 deficiency in mouse bladder superficial epithelium^14^. The role of autophagy in UPEC clearance remains unclear.

Lots of host factors responsible for sensing invasive pathogens and initiating anti-bacterial effects are induced during bacterial infections. It can be triggered through recognition of pathogen-associated molecular patterns (PAMPs) by a variety of pattern recognition receptors (PRRs), such as Toll-like receptors (TLRs)^15–17^. RhoB, a member of the small Rho GTPases family, is rapidly and transiently upregulated by cell exposure to LPS or inflammatory cytokines, which may be dependent on the NF-κB pathway^18–20^. RhoB contributes to multiple cellular functions, such as protein trafficking, maintaining endothelial barrier integrity, modulating cell migration and adhesion^18^, and induction of cytokines that regulate inflammatory events^21,22^. In addition, post-translational modification of RhoB promotes lysosomal translocation and degradation of mTORC1, resulting in an increase of DNA damage-induced autophagy^23^. Beclin 1 is the central component of PI3K complex and mainly responsible for assembling diverse regulatory factors that are involved in autophagy^24^. Expression of *Beclin 1* contributes to the induction of autophagy^25,26^. So far, the role and underlying mechanisms of RhoB in regulating Beclin 1 and bacterial clearance are unknown.

In this study, we found that UPEC induced RhoB in human bladder epithelial cells, which subsequently promoted clearance of intracellular bacteria. RhoB upregulation increased Beclin 1 stabilization and LC3 lipidation, both hallmarks of autophagy. Notably, we identified that RhoB physically bound to Beclin 1 and enhanced the association of Hsp90 with Beclin 1, resulting in LC3 lipidation and clearance of intracellular UPEC. The present study reveals the role of RhoB in defending against intracellular UPEC and highlights the importance of the physical association between RhoB, Beclin 1, and Hsp90 to achieve anti-bacterial effects.

## Results

### RhoB restricts intracellular UPEC

**Fig. 1:**
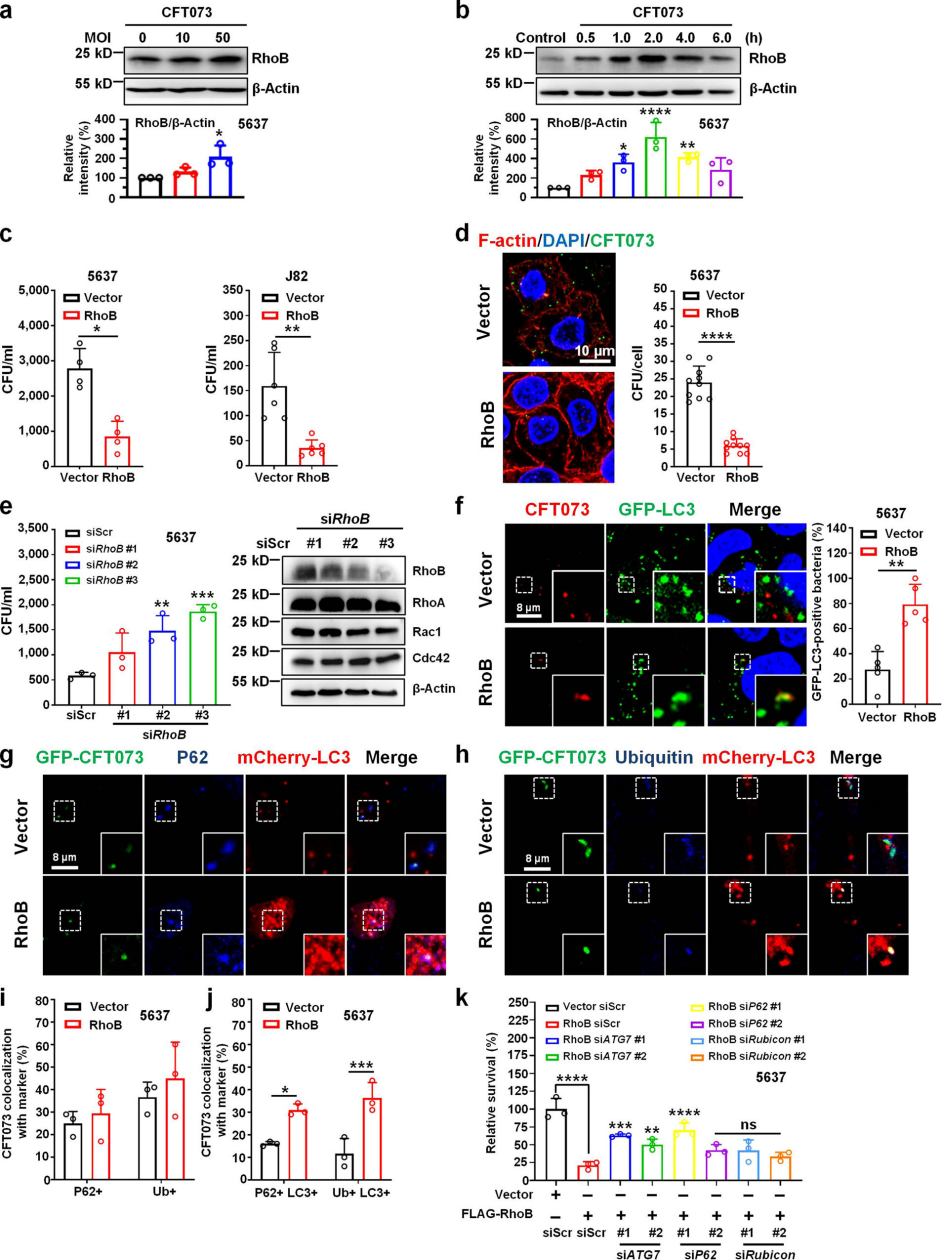
RhoB restricts intracellular UPEC. **a** RhoB level in 5637 bladder epithelial cells infected by UPEC at indicated MOIs. The RhoB density was normalized to that of β-Actin. The relative density of uninfected cells was set to 100%. (*n* = 3 independent experiments). MOI 0 vs. 10 *P* = 0.4998, MOI 0 vs. 50 *P* = 0.0191. **b** RhoB level in UPEC-infected 5637 cells at indicated time after infections. The RhoB density was normalized to that of β-Actin. The relative density of uninfected cells was set to 100%. (*n* = 3 independent experiments). Control vs. 0.5 *P* = 0.2944, control vs. 1.0 *P* = 0.0144, control vs. 2.0 *P* < 1.0e−15, control vs. 4.0 *P* = 0.0040, control vs. 6.0 *P* = 0.0909. **c** *RhoB* overexpression decreases intracellular UPEC in 5637 bladder epithelial cells or J82 cells at 2 hpi. Left panel: *n* = 4 independent experiments, *P* = 0.0286; right panel: *n* = 6 independent experiments, *P* = 0.0022. **d** Representative image of *RhoB*-overexpressing 5637 bladder epithelial cells infected by CFT073 at 4 hpi. *n* = 10 random areas assessed from three independent experiments (more than 100 cells for each group). Scale bar, 10 μm. *P* = 1.1e−5. **e** Knockdown of *RhoB* increases intracellular UPEC in 5637 bladder epithelial cells. Knockdown efficiency of *RhoB* siRNAs in 5637 cells was shown on the right. *n* = 3 independent experiments. siScr vs. si*RhoB* #1 *P* = 0.1299, siScr vs. si*RhoB* #2 *P* = 0.0070, siScr vs. si*RhoB* #3 *P* = 0.0007. **f** Representative confocal images and quantification of GFP-LC3-positive UPEC in *RhoB*-overexpressing 5637 bladder epithelial cells. *n* = 5 random areas in images of each group from three independent experiments. Scale bars, 8 μm. *P* = 0.0079. **g**–**h** Representative confocal images of 5637 cells infected with GFP-expressing CFT073 for 1 h in the presence of BafA1. Scale bars, 8 μm. **i** Quantification of P62- or Ubiquitin-positive UPEC in *RhoB*-overexpressing 5637 cells at 1 hpi. *n* = 3 independent experiments. P62 + *P* = 0.8627, Ub+ *P* = 0.5941. **j** Quantification of GFP-CFT073 colocalization with P62, Ubiquitin and mCherry-LC3 in 5637 cells at 1 hpi. *n* = 3 independent experiments. P62 + LC3 + *P* = 0.0121, Ub+ LC3 + *P* = 0.0006. **k** Knockdown of *ATG7* and *P62* restores bacterial survival in *RhoB*-overexpressing 5637 bladder epithelial cells. Bacterial survival of vector and scramble siRNA-transfected cells was set to 100%. Statistical significance was calculated by comparing the mean of each group with that of RhoB siScr group. *n* = 3 independent experiments. RhoB siScr vs. Vector siScr *P* < 1.0e−15, RhoB siScr vs. RhoB si*ATG7* #1 *P* = 0.0004, RhoB siScr vs. RhoB si*ATG7* #2 *P* = 0.0093, RhoB siScr vs. RhoB si*P62* #1 *P* < 1.0e−15, RhoB siScr vs. RhoB si*P62* #2 *P* = 0.0710, RhoB siScr vs. RhoB si*Rubicon* #1 *P* = 0.0770, RhoB siScr vs. RhoB si*Rubicon* #2 *P* = 0.4768. Data are shown as the mean ± SD, *P < 0.05, **P < 0.01, ***P < 0.001, ****P < 0.0001, two-tailed unpaired Student’s *t* test (**c**–**d**, **f**), one-way ANOVA (**a**–**b**, **e**, **k**), two-way ANOVA (**i**–**j**).

To explore the role of RhoB during UTIs, we first assessed the protein level of RhoB in human bladder epithelial cell line 5637 upon infection with the UPEC strain CFT073. Endogenous RhoB was markedly increased at 2 h post infection (hpi) in a MOI (multiplicity of infection)-dependent manner (Fig. 1a). RhoB was detected to be increased immediately at 0.5 hpi, significantly induced at 1 hpi, reach a peak at 2 hpi, and maintain till 4 hpi (Fig. 1b). To determine if LPS alone is sufficient to induce RhoB, we employed LPS treatment in 5637 bladder epithelial cells and observed that LPS gradually upregulated *RhoB* expression; whereas, it reached a peak at 2 h post-treatment (Supplementary Fig. 1a and b); which is consistent with an earlier report showing that *RhoB* expression is induced by LPS^19^. To explore the underlying functions of RhoB during UTIs, FLAG-tagged *RhoB* was transfected into 5637 bladder epithelial cells or another human bladder epithelial cell line (J82) for bacterial invasion assays. The number of intracellular bacteria from *RhoB*-transfected 5637 bladder epithelial cells, compared with that from vector-transfected cells, was reduced by 3-fold at 2 hpi without affecting bacterial entry (Fig. 1c and Supplementary Fig. 1c). Immunofluorescent staining revealed that the number of intracellular bacteria per cell was reduced in *RhoB* over-expressing cells (Fig. 1d and Supplementary Fig. 1d). Interestingly, a bacterial expulsion assay indicated that the number of extracellular CFT073 in the supernatant from *RhoB*-overexpressing cells was also decreased, compared with that from control cells (Supplementary Fig. 1e), suggesting that RhoB promotes bacterial clearance post bacterial entry. To further examine this possibility, endogenous *RhoB* was knocked down using specific siRNAs, which led to a threefold increase of intracellular bacteria, compared with that achieved by siScr control (Fig. 1e). These observations suggest that RhoB is induced by UPEC infection and plays a key role in decreasing intracellular UPEC.

Since autophagy plays an essential role in cell defense against bacterial infection^14,27,28^, we hypothesized that autophagy is involved in RhoB-mediated clearance of intracellular UPEC. We found that the level of LC3-II was increased in 5637 bladder epithelial cells infected with CFT073 in a MOI-dependent manner (Supplementary Fig. 1f), and significantly increased at 1 to 2 hpi (Supplementary Fig. 1g). Accumulation of LC3-II was also observed in LPS-treated cells (Supplementary Fig. 1h). To further verify this result, we transfected tandem-tagged mCherry-GFP-LC3 into 5637 bladder epithelial cells and treated them with CFT073 or LPS. As expected, a higher number of autophagosomes (Yellow puncta) and autolysosomes (Red puncta) were detected in CFT073 or LPS-treated cells, compared with that detected in control cells (Supplementary Fig. 1i–j).

Interestingly, we observed that intracellular CFT073 decorated by GFP-LC3 was increased in 5637 bladder epithelial cells when *RhoB* was overexpressed (Fig. 1f and Supplementary Fig. 2a). In addition, both P62-LC3 and Ubiquitin-LC3 double-positive CFT073 were detected to be increased in *RhoB* overexpressing cells (Fig. 1g–j and Supplementary Fig. 2b–c), and colocalization of CFT073 with P62 or Ubiquitin alone was not affected by RhoB (Fig. 1i). In addition, inhibitors of autophagic flux, such as ammonium chloride (NH_4_Cl) and bafilomycinA1 (BafA1), were applied in *RhoB*-overexpressing cells infected by CFT073. Of note, both inhibitors efficiently restored the number of intracellular bacteria reduced by overexpression of *RhoB* (Supplementary Fig. 2d). We also knocked down *ATG7*, *P62,* or *Rubicon* using siRNAs, to specifically block biogenesis of autophagosomes or LC3-associated phagocytosis (LAP) and found that efficient knockdown of *ATG7* or *P62* abolished RhoB-mediated clearance of intracellular UPEC, and *Rubicon* knockdown did not attenuate the effect of RhoB (Fig. 1k and Supplementary Fig. 2e–g). It is likely that RhoB promotes intracellular UPEC clearance through autophagy-related pathways.

### RhoB induces LC3 lipidation and UPEC clearance through Beclin1

**Fig. 2:**
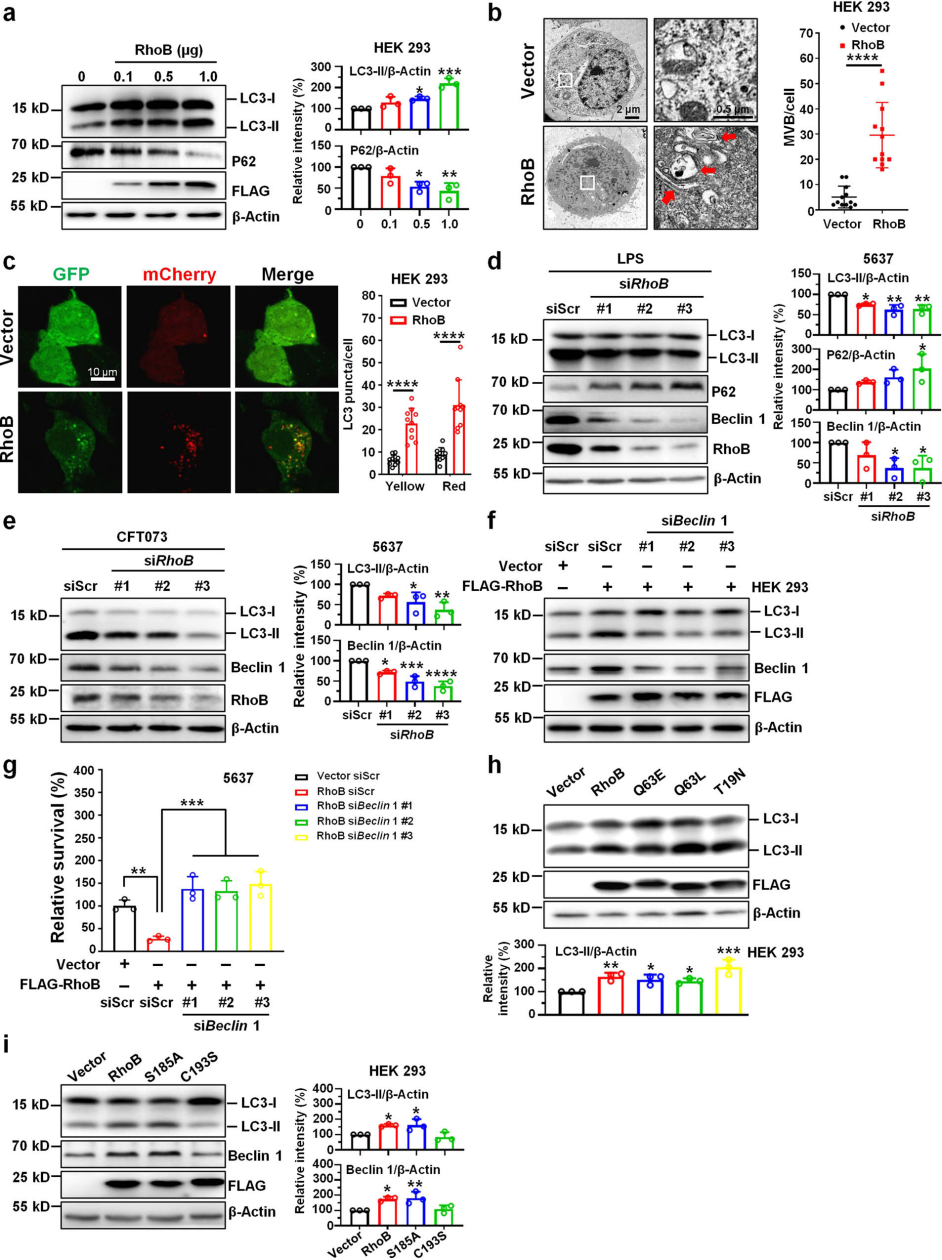
RhoB induces LC3 lipidation and upregulates Beclin1 level. **a** Protein levels of LC3 and P62 in HEK293 cells transfected with *RhoB* or vector. Signal densities of RhoB and P62 were normalized to that of β-Actin. The relative density of vector-transfected cells was set to 100%. *n* = 3 independent experiments. LC3-II/β-Actin: 0 vs. 0.1 *P* = 0.1768, 0 vs. 0.5 *P* = 0.0306, 0 vs. 1.0 *P* = 0.0001; P62/β-Actin: 0 vs. 0.1 *P* = 0.2468, 0 vs. 0.5 *P* = 0.0103, 0 vs. 1.0 *P* = 0.0031. **b** TEM images of *RhoB* overexpressing cells. Quantification on the right shows the number of autophagic vacuoles per cell (*n* = 12 cells per group from three independent experiments). Arrows show autophagic vacuoles. Scale bars, 2 or 0.5 μm. *P* = 7.4e−7. **c** Representative images of HEK 293 cells transfected with mCherry-GFP-LC3 in the presence of RhoB or vector. Autophagosomes, yellow puncta; autolysosomes, red-only puncta. Bar graph shows the quantification of LC3 puncta per cell (*n* = 10 random areas per group from three independent experiments). Scale bar, 10 μm. Yellow *P* = 1.1e−5, Red *P* = 3.2e−8. **d**, **e** Western blotting showing the levels of autophagic proteins in *RhoB* knockdown 5637 bladder epithelial cells with LPS (**d**) or with CFT073 infection (**e**). 5637 bladder epithelial cells were transfected with *RhoB* siRNAs or scramble siRNA for 48 h followed by infection of CFT073 at MOI of 50 for 2 h. Signal densities of LC3-II, P62, and Beclin 1 were normalized to that of β-Actin. The relative density of siScr-transfected cells was set to 100%. *n* = 3 independent experiments. **d** LC3-II/β-Actin: siScr vs. si*RhoB* #1 *P* = 0.0133, siScr vs. si*RhoB* #2 *P* = 0.0011, siScr vs. si*RhoB* #3 *P* = 0.0013; P62/β-Actin: siScr vs. si*RhoB* #1 *P* = 0.5419, siScr vs. si*RhoB* #2 *P* = 0.2053, siScr vs. si*RhoB* #3 *P* = 0.0290; Beclin 1/β-Actin: siScr vs. si*RhoB* #1 *P* = 0.3483, siScr vs. si*RhoB* #2 *P* = 0.0400, siScr vs. si*RhoB* #3 *P* = 0.0390. **e** LC3-II/β-Actin: siScr vs. si*RhoB* #1 *P* = 0.1356, siScr vs. si*RhoB* #2 *P* = 0.0225, siScr vs. si*RhoB* #3 *P* = 0.0030; Beclin 1/β-Actin: siScr vs. si*RhoB* #1 *P* = 0.0131, siScr vs. si*RhoB* #2 *P* = 0.0004, siScr vs. si*RhoB* #3 *P* < 1.0e−15. **f** Knockdown of *Beclin 1* restores LC3 lipidation in *RhoB*-overexpressing HEK 293 cells. Three independent experiments. **g** Knockdown of *Beclin 1* restores bacterial survival in *RhoB*-overexpressing 5637 bladder epithelial cells. 5637 cells were co-transfected with *RhoB* and *Beclin 1* siRNAs for 48 h followed by bacterial invasion assay. Bacterial survival of vector and siScr-transfected cells was set to 100%. *n* = 3 independent experiments. RhoB siScr vs. Vector siScr *P* = 0.0062, RhoB siScr vs. RhoB si*Beclin 1* #1 *P* = 0.0003, RhoB siScr vs. si*Beclin 1* #2 *P* = 0.0004, RhoB siScr vs. si*Beclin 1* #3 *P* = 0.0002. **h** LC3 lipidation in HEK293 cells transfected with plasmids encoding RhoB-WT, Q63E, Q63L, and T19N. The LC3-II density was normalized to that of β-Actin. The relative density of vector-transfected cells was set to 100%. *n* = 3 independent experiments. LC3-II/β-Actin: Vector vs. RhoB *P* = 0.0069, Vector vs. Q63E *P* = 0.0249, Vector vs. Q63L *P* = 0.0480, Vector vs. T19N *P* = 0.0002. **i** LC3 lipidation and Beclin 1 level in HEK293 cells transfected with plasmids encoding RhoB-WT, S185A, and C193S for 48 h. Signal densities of LC3-II and Beclin 1 were normalized to that of β-Actin. The relative density of vector-transfected cells was set to 100%. *n* = 3 independent experiments. LC3-II/β-Actin: Vector vs. RhoB *P* = 0.0459, Vector vs. S185A *P* = 0.0398, Vector vs. C193S *P* = 0.7997; Beclin 1/β-Actin: Vector vs. RhoB *P* = 0.0136, Vector vs. S185A *P* = 0.0092, Vector vs. C193S *P* = 0.9421. Data are the mean ± SD, *P < 0.05, **P < 0.01, ***P < 0.001, ****P < 0.0001, two-tailed unpaired Student’s *t* test (**b**), one-way ANOVA (**a**, **d**–**e**, **g**–**i**), or two-way ANOVA (**c**).

We detected changes of autophagic proteins in HEK293 cells that overexpressed *RhoB*. Interestingly, RhoB dosage-dependently accelerated the processing of LC3 lipidation, shown as an accumulation of LC3-II (Fig. 2a), which was also observed in human prostate epithelial PC-3 cells (Supplementary Fig. 3a). The level of P62, a marker protein of autophagic degradation^29^, was conversely reduced by *RhoB* overexpression (Fig. 2a). Transmission electron microscope (TEM) revealed more autophagic vacuoles in the cytosol of *RhoB*-overexpressing cells, in stark contrast to those in control cells (Fig. 2b). The number of LC3 puncta (both yellow and red) in *RhoB*-overexpressing cells was also threefold higher than that in control cells (Fig. 2c). In line with these results, knockdown of *RhoB* in 5637 bladder epithelial cells mitigated LC3 lipidation and led to an accumulation of P62 (Fig. 2d). Interestingly, treatment with BafA1 also led to an increase of LC3-II in *RhoB*-overexpressing cells (Supplementary Fig. 3b), reflecting that RhoB likely promotes autophagic flux rather than blocking lysosomal degradation. Notably, the decrease of Beclin 1, the core adaptor of PI3K complex that mediates autophagosome initiation, was observed when *RhoB* was knocked down (Fig. 2d). This reduction was validated in CFT073-infected 5637 bladder epithelial cells (Fig. 2e), and increased Beclin 1 was observed in both *RhoB*-transfected T24 bladder epithelial cells and LPS-pretreated 5637 bladder epithelial cells (Supplementary Fig. 3c and d). Moreover, knockdown of *Beclin 1* in *RhoB*-overepxressing cells restored LC3-II protein level and intracellular bacteria survival (Fig. 2f–g and Supplementary Fig. 3e), suggesting that RhoB affects autophagic protein LC3 lipidation and UPEC clearance through Beclin 1.

We next investigated whether activation of RhoB affected its activity in LC3 lipidation. Constitutively active (Q63E and Q63L) and dominant-defective (T19N) constructs of RhoB were generated and validated using the Rhotekin pull-down assay (Supplementary Fig. 3f). No significant difference was observed between the effects of Q63L, Q63E, T19N, and wild-type RhoB on LC3 lipidation (Fig. 2h), suggesting that RhoB activation is not important toward its role in LC3 lipidation.

Posttranslational modifications of RhoB at its C-terminal region are known to be functional-related^30^. Geranylgeranylation and farnesylation of RhoB at cysteine 193 of its C-terminal CAAX motif is critical for its membrane-associated localization (including the plasma membrane, endosomes, and multivesicular bodies) and biological activities^18^. Phosphorylation of RhoB at serine 185 affects its functions in cytoskeleton organization^31^. To abrogate its posttranslational modifications, we selectively made two substitutions of RhoB, RhoB-S185A, and RhoB-C193S. Using immunofluorescent staining, we observed that wild-type RhoB and RhoB-S185A were similar, while subcellular location of RhoB-C193S was altered, similar as reported previously^30^ (Supplementary Fig. 3g). Overexpression of *RhoB*-C193S in 293 cells impeded LC3 lipidation and upregulation of Beclin 1, whereas RhoB-S185A retained similar effects as the wild-type (Fig. 2i). These observations demonstrate that the activity of RhoB in LC3 lipidation relies on its localization with membrane.

As RhoB was induced by LPS, we examined whether TLR4 (receptor of LPS) is involved in LPS-mediated RhoB upregulation. *TLR4* knockdown using siRNA in 5637 bladder epithelial cells greatly reduced RhoB, Beclin 1, and LC3-II levels stimulated by LPS (Supplementary Fig. 3h).

### RhoB physically interacts with Beclin1 and prevents its degradation

In order to elucidate the underlying mechanism through which RhoB affects Beclin 1 level, we examined RhoB-induced changes of *Beclin 1* mRNA transcription and protein degradation. No significant change was observed in *Beclin1* mRNA level when *RhoB* was ectopically expressed in 5637 bladder epithelial cells (Supplementary Fig. 4a). To evaluate the effect of RhoB on Beclin 1 protein degradation, *RhoB*-transfected HEK293 cells (or vector control-transfected cells) were treated with cycloheximide (CHX), an inhibitor of protein synthesis. The level of MYC-Beclin 1 was strongly decreased in vector-transfected HEK293 cells in the presence of CHX, whereas the level of MYC-Beclin 1 was still increased in *RhoB*-overexpressing cells treated by CHX, suggesting that RhoB does not affect Beclin 1 synthesis but may prevent the Beclin 1 degradation (Supplementary Fig. 4b). To confirm this, endogenous *RhoB* in 5637 bladder epithelial cells was knocked down using siRNAs and the cells were treated with the proteasomal inhibitor MG-132 to block Beclin 1 degradation simultaneously. The level of endogenous Beclin 1 was markedly decreased without MG-132 treatment in *RhoB* knocked down cells, but not in control cells; however, no decrease of Beclin 1 was found in *RhoB* knocked down cells treated with MG-132 (Supplementary Fig. 4c). These results indicate that RhoB increases Beclin 1 level via blockage of its degradation, and RhoB has no effect on Beclin 1 transcription and synthesis.

**Fig. 3:**
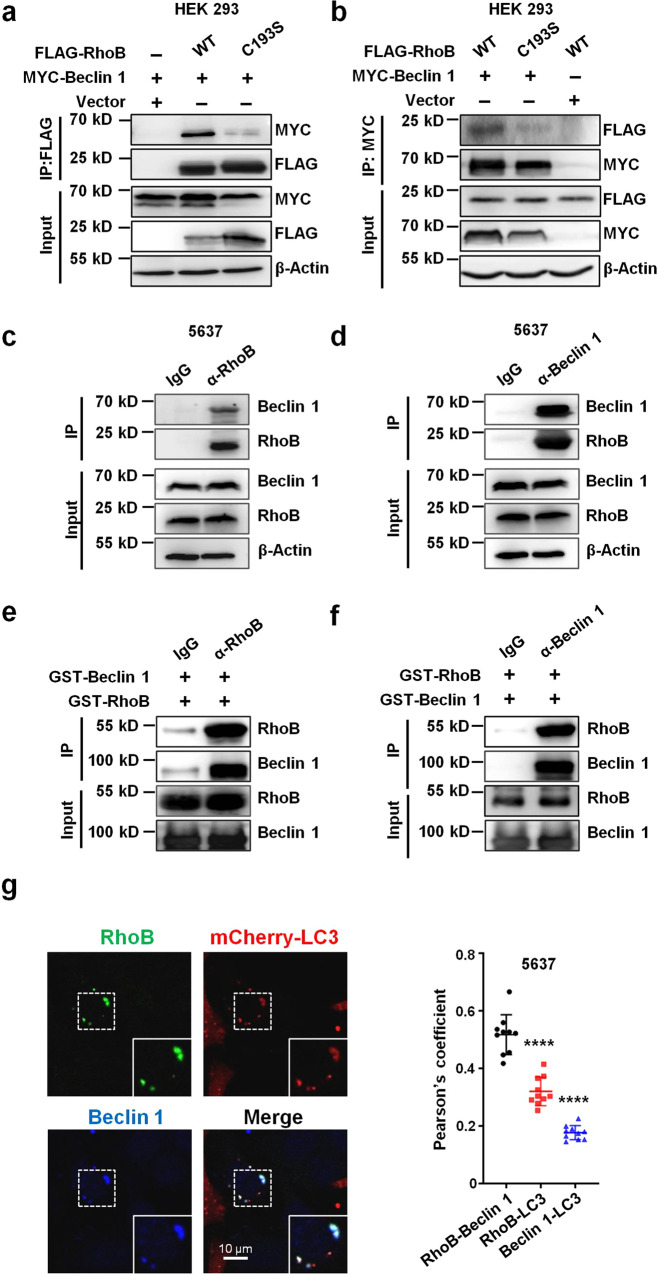
RhoB physically interacts with Beclin1 and prevents its degradation. **a**, **b** The interaction of overexpressed RhoB with Beclin 1 in HEK 293 cells. HEK 293 cells were co-transfected with MYC-Beclin 1, FLAG-RhoB WT, FLAG-RhoB-C193S or Vector. At 48 h post transfection, cell lysates were prepared and subjected to immunoprecipitation with anti-FLAG (**a**) or anti-MYC (**b**) antibodies followed by western blotting analysis. *n* = 3 independent experiments. **c**, **d** Reciprocal immunoprecipitation of endogenous Beclin 1 and RhoB in 5637 bladder epithelial cells. 5637 cells were treated with LPS for 2 h to upregulate *RhoB* expression and lysed. Cell lysates were immunoprecipitated with anti-RhoB (**c**) or anti-Beclin 1 (**d**) antibodies and analyzed by western blotting. The slanting labels identify the IP antibodies. IgG served as a negative control. Three independent experiments. **e**, **f** RhoB directly interacts with Beclin 1. GST-fused RhoB and Beclin 1 were expressed in *E. coli* and subjected to GST pull-down assay with anti-RhoB (**e**) or anti-Beclin 1 (**f**) antibodies. The slanting labels identify the IP antibodies. IgG served as a negative control. *n* = 3 independent experiments. **g** Colocalization of endogenous RhoB and Beclin 1 in mCherry-*LC3*-expressing in CFT073-infected 5637 cells (left). 5637 cells were transfected with mCherry-LC3 and infected with CFT073 for 2 h, followed by immunostaining with Alexa Fluor 488-conjugated anti-RhoB antibody (green), anti-Beclin 1 antibody plus Alexa Fluor 405-conjugated secondary antibody (blue). Quantification of colocalization was shown by calculating the Pearson correlation coefficients (*R* values) using Image Pro Plus software (right). *n* = 10 random areas per group from three independent experiments. Scale bar, 10 μm. RhoB-Beclin 1 vs. RhoB-LC3 *P* < 1.0e−15, RhoB-Beclin 1 vs. Beclin 1-LC3 *P* < 1.0e−15. Pearson’s coefficients for RhoB-Beclin 1, RhoB-LC3 and Beclin 1-LC3 colocalization were ~0.52, ~0.32, and ~0.18, respectively. Data are the mean ± SD, ****P < 0.0001, one-way ANOVA.

RhoB acts as an intracellular transporter that mediates trafficking events of cellular factors through protein-protein interactions. This activity regulates many cellular functions, such as integrity of endothelial barrier, cell migration, and adhesion, as well as cancer development^18,20,32–34^. Therefore, we hypothesized that RhoB increases Beclin 1 stabilization through directly interacting with Beclin 1. To evaluate this, we carried out co-immunoprecipitation assays using HEK293 cells co-transfected with MYC-tagged *Beclin 1*, FLAG-tagged *RhoB*-WT or *RhoB*-C193S. Intriguingly, Beclin 1 was efficiently co-immunoprecipitated with RhoB-WT, but not with vector or RhoB-C193S, and vice versa (Fig. 3a and b). This RhoB-Beclin 1 association was reciprocally verified by co-immunoprecipitation with endogenous proteins from 5637 bladder epithelial cells (Fig. 3c and d). Purified GST-fused RhoB and Beclin 1 were next applied in immunoprecipitation assays, to determine if RhoB directly binds to Beclin 1. As expected, GST-Beclin 1 with anti-Beclin 1 antibody, but not with the IgG control, was found to efficiently pull down GST-RhoB, and vice versa (Fig. 3e and f). Moreover, colocalization of endogenous RhoB and Beclin 1 was observed with LC3 puncta (Fig. 3g and Supplementary Fig. 4d, Pearson’s coefficients for RhoB-Beclin 1, RhoB-LC3 and Beclin 1-LC3 colocalization were ~0.52, ~0.32, and ~0.18, respectively), reflecting that RhoB and Beclin 1 might form a complex to maintain Beclin 1 for autophagosome formation. Taken together, these data reveal that RhoB binds to Beclin 1 directly and maintains Beclin 1 level by attenuating its degradation. Colocalization of endogenous RhoB and Beclin 1 was also observed in the cytoplasm of 5637 bladder epithelial cell infected by CFT073, and less colocalization of RhoB with CFT073 was detected (Supplementary Fig. 4e, Pearson’s coefficients for RhoB-Beclin 1, RhoB-CFT073 and Beclin 1-CFT073 colocalization were ~0.45, ~0.06, and ~0.19, respectively).

### Molecular interactions between RhoB and Beclin 1

**Fig. 4:**
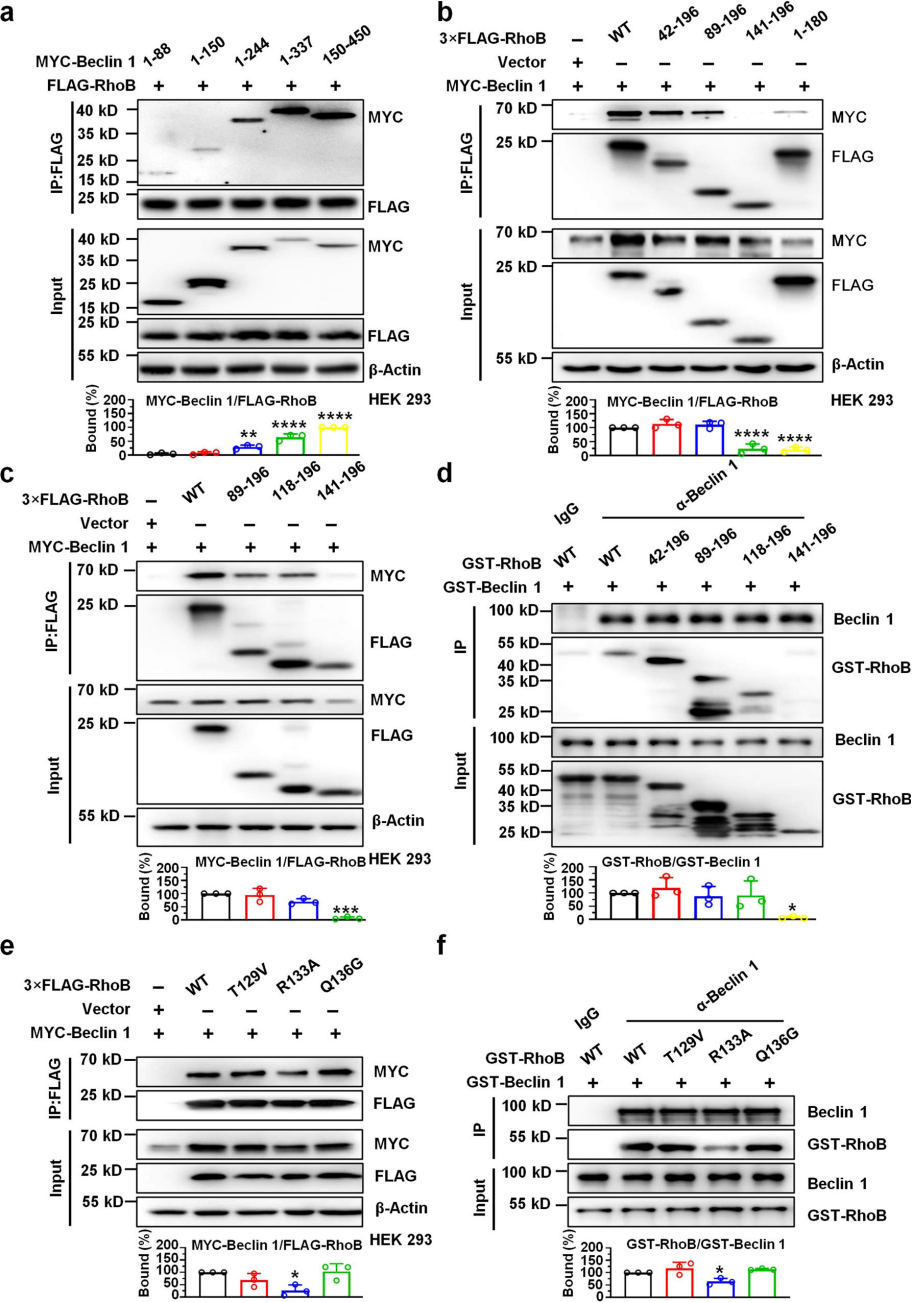
Molecular interactions between RhoB and Beclin 1. **a**, **b** Mapping the interacting regions between RhoB and Beclin 1. HEK 293 cells were co-transfected with truncation mutants of MYC-Beclin 1 and 3×FLAG-RhoB (**a**), or reciprocally with truncation mutants of 3×FLAG-RhoB and MYC-Beclin 1 (**b**), followed by immunoprecipitation and western blotting analysis. The density of MYC-Beclin 1 was normalized to that of FLAG-RhoB in IP group. In Fig. 4a, the percentage of bound MYC-Beclin 1 150–450 was set to 100%. In Fig. 4b, the percentage of bound MYC-Beclin 1 to RhoB WT was set to 100%. *n* = 3 independent experiments. **a** MYC-Beclin 1/FLAG-RhoB: 1–88 vs. 1–150 *P* = 0.9771, 1–88 vs. 1–244 *P* = 0.0033, 1–88 vs. 1–337 *P* < 1.0e−15, 1–88 vs. 150–450 *P* < 1.0e−15; **b** MYC-Beclin 1/FLAG-RhoB: WT vs. 42–196 *P* = 0.5048, WT vs. 89–196 *P* = 0.6684, WT vs. 141–196 *P* < 1.0e−15, WT vs. 1–180 *P* < 1.0e−15. **c** Interaction of Beclin 1 with RhoB truncation mutants at helical region. The density of MYC-Beclin 1 was normalized to that of FLAG-RhoB in IP group. The percentage of bound MYC-Beclin 1 to RhoB WT was set to 100%. *n* = 3 independent experiments. MYC-Beclin 1/FLAG-RhoB: WT vs. 89–196 *P* = 0.9445, WT vs. 118–196 *P* = 0.0680, WT vs. 141–196 *P* = 0.0001. **d** GST pull-down assay with GST-fused Beclin 1 and RhoB truncation mutants. The slanting labels identify the IP antibodies. IgG served as a negative control. The density of GST-RhoB was normalized to that of GST-Beclin 1 in IP group. The percentage of bound GST-RhoB WT to GST-Beclin 1 was set to 100%. *n* = 3 independent experiments. GST-RhoB/GST-Beclin 1: WT vs. 42–196 *P* = 0.8909, WT vs. 89–196 *P* = 0.9759, WT vs. 118–196 *P* = 0.9924, WT vs. 141–196 *P* = 0.0257. **e** Mapping the key binding site of RhoB-Beclin 1 interaction at helical region of RhoB. The density of MYC-Beclin 1 was normalized to that of FLAG-RhoB in IP group. The percentage of bound MYC-Beclin 1 to RhoB WT was set to 100%. *n* = 3 independent experiments. MYC-Beclin 1/FLAG-RhoB: WT vs. T129V *P* = 0.3089, WT vs. R133A *P* = 0.0133, WT vs. Q136G *P* = 0.9961. **f** GST pull-down assay with GST-fused Beclin 1 and RhoB single mutants at helical region. The slanting labels identify the IP antibodies. IgG served as a negative control. The density of GST-RhoB was normalized to that of GST-Beclin 1 in IP group. The percentage of bound GST-RhoB WT to GST-Beclin 1 was set to 100%. *n* = 3 independent experiments. GST-RhoB/GST-Beclin 1: WT vs. T129V *P* = 0.3277, WT vs. R133A *P* = 0.0305, WT vs. Q136G *P* = 0.5267. Data are the mean ± SD, *P < 0.05, **P < 0.01, ***P < 0.001, ****P < 0.0001, one-way ANOVA (**a**–**f**).

Three functional domains of Beclin 1, including Bcl-2-homology-3 (BH3)-only domain, central coiled-coiled domain (CCD), and evolutionary conserved domain (ECD) have been identified. In order to map the interface of RhoB-Beclin 1 interaction in detail, a series of truncation mutants of MYC-Beclin1 and FLAG-RhoB were genetically engineered. Co-immunoprecipitation assay with MYC-Beclin 1 constructs and FLAG-RhoB indicated that MYC-Beclin 1 truncation mutants including 1–244, 1–337, and 150–450, but not 1–88 and 1–150, were able to bind to FLAG-RhoB (Fig. 4a and Supplementary Fig. 5a). In addition, GST-Beclin 1 truncation mutants (88–450, 150–450, 244–450, and 337–450) were constructed for GST pull-down analysis, and the interactions between RhoB and two Beclin 1 truncation mutants (88–450 and 150–450) could be detected. These results suggest that the CCD domain (150–244) of Beclin 1 is necessary for RhoB-Beclin 1 interaction (Supplementary Fig. 5b). Reciprocally, co-immunoprecipitation assay with FLAG-RhoB constructs and MYC-Beclin 1 demonstrated that residues (89–140) of RhoB are indispensable for their interaction (Fig. 4b and Supplementary Fig. 5c). These RhoB constructs, such as RhoB-WT and RhoB (89–196), that interacted with Beclin 1 coordinately enhanced Beclin 1 stabilization and LC3 lipidation, supporting the idea that binding of RhoB to Beclin 1 is essential for the activity of RhoB in mediating LC3 lipidation (Supplementary Fig. 5d and e).

Based on structural information obtained from the Protein Data Bank (RhoB, 2FV8), we noticed that RhoB contains a helical region at residues (118–134) that might be crucial for its interaction with Beclin 1 (Supplementary Fig. 5f). Therefore, we additionally generated truncation mutant RhoB (118–196) to find out the exact region of RhoB-Beclin 1 interaction. Co-immunoprecipitation and GST pull-down results revealed that the helical region located in residues (118–140) is required for RhoB-Beclin 1 interaction (Fig. 4c and d). Based on RhoB structure, we hypothesized two amino acids (T129, R133) in this helical region, which contain side-chain groups that may form hydrogen bonds with other proteins, are involved in RhoB-Beclin 1 interaction. Therefore, substitutions of RhoB, T129V, R133A, and Q136G (located in the adjacent loop region, used as the negative control), were constructed for co-immunoprecipitation and GST pull-down analysis. Notably, R133A attenuated the interaction between RhoB and Beclin 1 compared with RhoB-WT, Q136G and T129V (Fig. 4e and f). This disruption of Beclin 1 interaction by RhoB-R133A led to a decrease of Beclin 1 level and coordinately reduced LC3 lipidation (Supplementary Fig. 5g and h). In addition, R133A did not significantly decrease intracellular bacterial survival compared with RhoB-WT, Q136G, and T129V (Supplementary Fig. 5i)

Collectively, these results suggest that RhoB physically interacts with the CCD domain of Beclin 1 by its residues (118–140), whereas Arg133 of RhoB might be the key binding site for RhoB-Beclin 1 interaction (Supplementary Fig. 5j).

### RhoB-mediated Beclin 1 stabilization and LC3 lipidation is governed by Hsp90

**Fig. 5:**
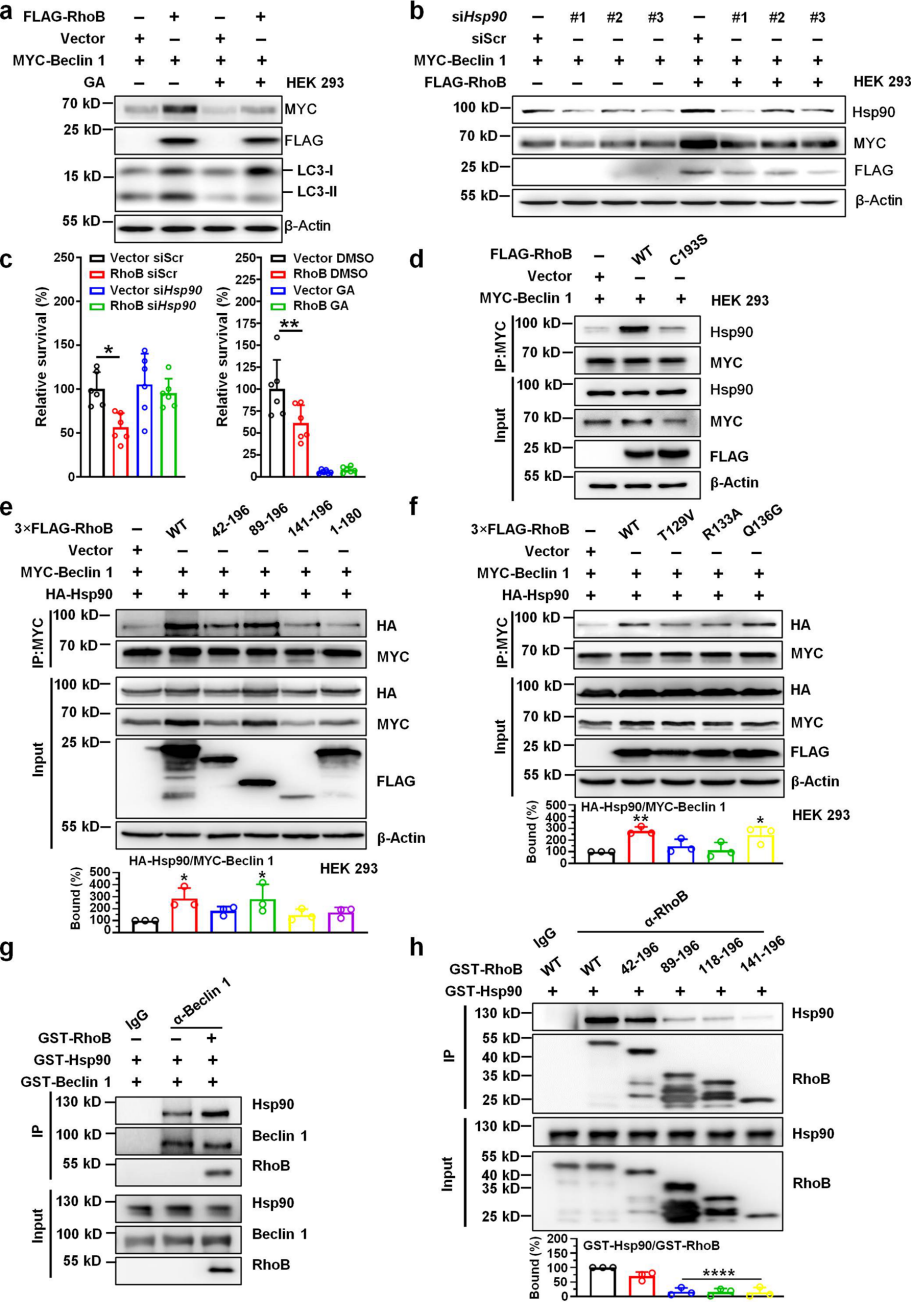
RhoB-mediated Beclin 1 stabilization is governed by Hsp90. **a** Protein level of Beclin 1 and LC3 in *RhoB*-overexpressing HEK293 cells in the presence or absence of Geldanamycin (GA, 1 μM). HEK 293 cells were transfected with MYC-Beclin 1 and FLAG-RhoB for 48 h, followed by treatment of GA overnight. *n* = 3 independent experiments. **b** Effect of *Hsp90* knocked down by siRNAs on the protein level of Beclin 1 in *RhoB*-overexpressing cells. *n* = 3 independent experiments. **c** Effects of *Hsp90* knocked down by siRNAs (left) or GA (right) on survival of intracellular CFT073 in *RhoB*-overexpressing cells. 5637 cells were co-transfected with FLAG-*RhoB* and *Hsp90* siRNAs followed by bacterial invasion assay. For the effect of GA, 5637 cells were transfected with FLAG-RhoB or vector in the presence or absence of GA, followed by bacterial invasion assay. Bacterial survival in Vector siScr or Vector DMSO groups was set to 100%. *n* = 6 independent experiments. Data are the mean ± SD, *P < 0.05. Left panel: Vector siScr vs. RhoB siScr *P* = 0.0100, Vector si*Hsp90* vs. RhoB si*Hsp90 P* = 0.7789; right panel: Vector DMSO vs. RhoB DMSO *P* = 0.0069, Vector GA vs. RhoB GA *P* = 0.9936. **d** The interaction of endogenous Hsp90 with Beclin 1 in *RhoB*-overexpressing cells. *n* = 3 independent experiments. **e**, **f** The interaction of Hsp90 with Beclin 1 in HEK 293 cells transfected with RhoB truncation mutants (**e**) and singl**e** mutants at helical region (**f**). HEK 293 cells were co-transfected with MYC-Beclin 1, HA-Hsp90 and indicated 3×FLAG-RhoB mutants followed by immunoprecipitation assay with anti-MYC antibody and western blotting analysis. The density of HA-Hsp90 was normalized to that of MYC-Beclin 1 in IP group. The percentage of bound HA-Hsp90 to MYC-Beclin 1 in RhoB WT group was set to 100%. *n* = 3 independent experiments. **e** HA-Hsp90/MYC-Beclin 1: Vector vs. WT *P* = 0.0257, Vector vs. 42–196 *P* = 0.4885, Vector vs. 89–196 *P* = 0.0304, Vector vs. 141–196 *P* = 0.8694, Vector vs. 1–180 *P* = 0.6351. **f** HA-Hsp90/MYC-Beclin 1: Vector vs. WT *P* = 0.0067, Vector vs. T129V *P* = 0.6605, Vector vs. R133A *P* = 0.9938, Vector vs. Q136G *P* = 0.0248. **g** GST pull-down assay with bacterially expressed GST-fused Beclin 1, Hsp90, and RhoB. Immunoprecipitation was performed with anti-Beclin 1 antibody or IgG. *n* = 3 independent experiments. **h** GST pull-down assay with bacterial expressed GST-fused Hsp90 and truncated RhoB proteins. Immunoprecipitation was performed with an anti-RhoB antibody or IgG. The density of GST-Hsp90 was normalized to that of GST-RhoB in IP group. The percentage of bound GST-Hsp90 to GST-RhoB WT was set to 100%. *n* = 3 independent experiments. GST-Hsp90/GST-RhoB: WT vs. 42–196 *P* = 0.0509, WT vs. 89–196 *P* < 1.0e−15, WT vs. 118–196 *P* < 1.0e−15, WT vs. 141–196 *P* < 1.0e−15. Data are the mean ± SD, *P < 0.05, **P < 0.01, ****P < 0.0001, one-way ANOVA (**c**, **e**–**f**, **h**).

Given that varieties of binding partners of Beclin 1 manipulate its degradation through ubiquitin machinery, we next examined the effect of RhoB on Beclin 1 ubiquitination. We found that both K11- and K48-linked ubiquitination of Beclin 1 were similar in *RhoB*-overexpressing and control cells (Supplementary Fig. 6a), indicating that there is another mechanism through which RhoB inhibits Beclin 1 degradation. Beclin 1 has been shown to be maintained by binding of Hsp90^35^. To determine whether RhoB-mediated Beclin 1 stabilization was associated with the stabilizing activity of Hsp90, we employed the specific inhibitor of Hsp90, Geldanamycin (GA), in our following experiments. Results showed that GA treatment, attenuated RhoB-induced LC3 lipidation and MYC-Beclin 1 stabilization (Fig. 5a). Similar results were obtained by *Hsp90* knocked down with siRNAs, which abrogated the upregulation of MYC-Beclin 1 induced by RhoB (Fig. 5b). In line with this, both knockdown of *Hsp90* and GA inhibition restored the diminished survival of intracellular CFT073 in *RhoB*-overexpressing 5637 bladder epithelial cells (Fig. 5c). No difference for Beclin 1 ubiquitination in *RhoB* overexpressing and control cells treated by GA was detected (Supplementary Fig. 6a). Taken together, these results support that Hsp90 is indispensable in increasing Beclin 1, LC3 lipidation, and bacterial clearance induced by RhoB.

We further examined if RhoB contributed to Beclin 1-Hsp90 interaction, resulting in decreased Beclin 1 degradation. Co-immunoprecipitation assay in HEK293 cells transfected with MYC-*Beclin 1* and FLAG-*RhoB* showed that RhoB-WT, but not RhoB-C193S, enhanced the binding of endogenous Hsp90 to MYC-Beclin 1 (Fig. 5d). Immunofluorescent assays indicated increased colocalization of Hsp90 with Beclin 1 in *RhoB*-overexpressing cells (Pearson’s coefficient, ~0.85), compared with that in control cells (Pearson’s coefficient, ~0.69, Supplementary Fig. 6b). Co-immunoprecipitation assay in HEK293 cells transfected with MYC-*Beclin 1*, HA-*Hsp90* and truncation mutants of FLAG-*RhoB* showed that RhoB-WT and RhoB (89–196) promoted the association of HA-Hsp90 with MYC-Beclin 1; yet, RhoB (42–196) did not obviously enhance the Beclin 1-Hsp90 interaction, in accordance with the above results showing that RhoB (42–196) did not promote LC3 lipidation (Fig. 5e, Supplementary Fig. 5d and e). RhoB-R133A, which attenuates the binding of RhoB to Beclin 1, was also observed to disrupt the Beclin 1-Hsp90 interaction (Fig. 5f). In GST pull-down assays, Beclin 1-Hsp90 interaction was enhanced in the presence of RhoB-WT (Fig. 5g). Collectively, these observations suggest that binding of RhoB to Beclin 1 enhances the Beclin 1-Hsp90 interaction, resulting in the blockage of Beclin 1 degradation and enhancement of LC3 lipidation.

Interestingly, endogenous RhoB was observed to be decreased in *Hsp90* knockdown cells, and overexpression of *Hsp90* significantly increased the protein level of endogenous RhoB (Supplementary Fig. 6c), suggesting that RhoB may also be a client protein of Hsp90. Co-immunoprecipitation assay with overexpressed truncation mutants of FLAG-*RhoB* and HA-*Hsp90* indicated that RhoB interacted with Hsp90 through RhoB residues 42–89 (Supplementary Fig. 6d), which is different from the interaction between RhoB and Beclin 1. This association was further validated by GST pull-down assay (Fig. 5h). Consistently, colocalization of RhoB with Hsp90 was observed in transfected HEK293 cells (Pearson’s coefficient, ~0.73, Supplementary Fig. 6e). In addition, we examined the interactions between RhoB, Beclin 1, and Hsp90 in 5637 bladder epithelial cells infected by CFT073. Induced RhoB and Beclin 1 were observed when cells were infected by CFT073, and more Beclin 1 was obtained by co-immunoprecipitation, binding with more Hsp90 and RhoB (Supplementary Fig. 6f). Overall, these data indicate that RhoB interacts with Beclin 1 and Hsp90 to form a RhoB-Beclin 1-Hsp90 complex (Supplementary Fig. 6g).

### RhoB promotes bacterial clearance in vivo

**Fig. 6:**
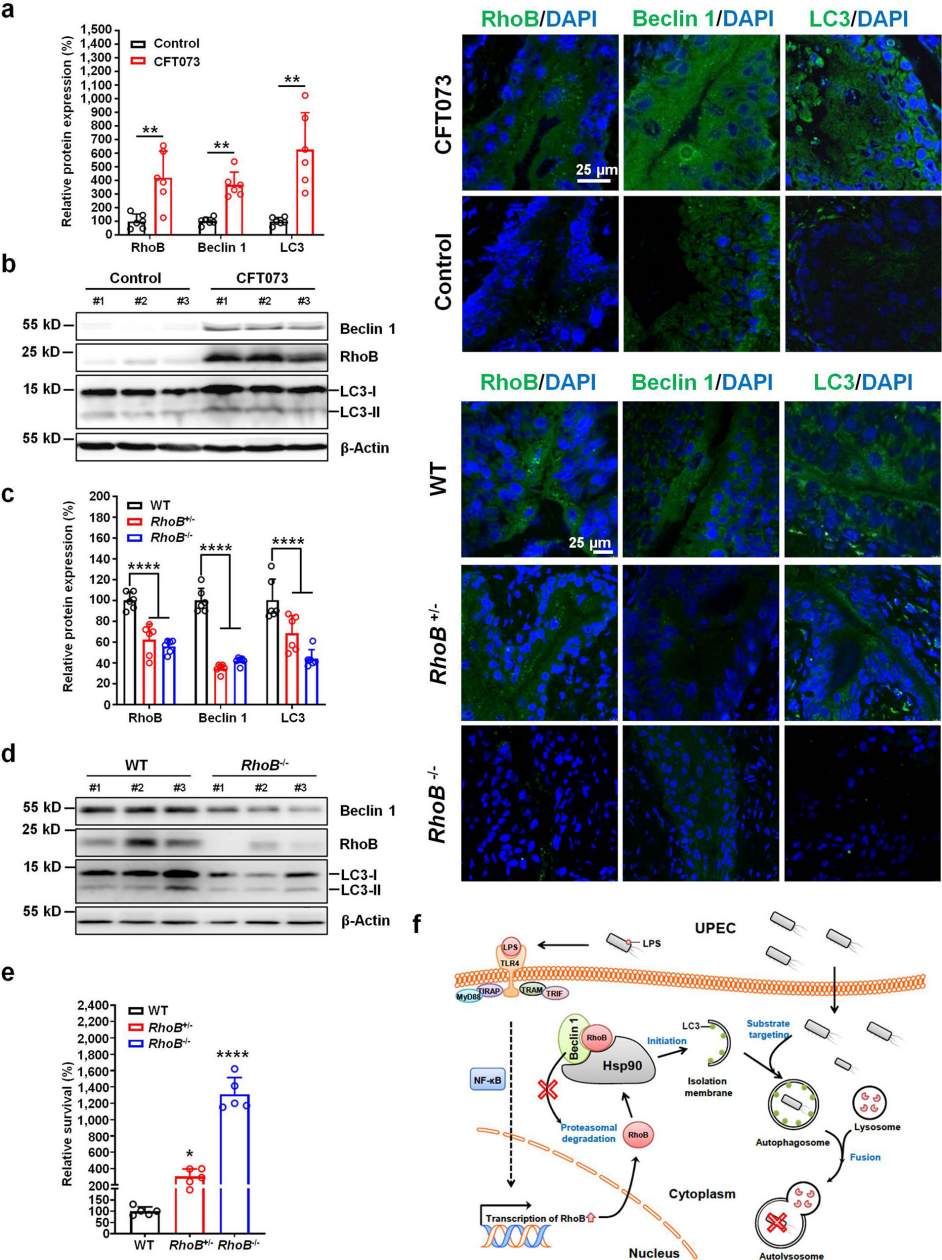
RhoB promotes bacterial clearance in vivo. **a**, **b** Protein level of RhoB, Beclin 1 and LC3 in CFT073-infected wild-type mice. Female C57BL/6J mice were transurethrally inoculated with 10^8^ CFU of CFT073 cultured overnight. **a** Immunohistofluorescence analysis. Levels of indicated proteins were quantified by measurement of fluorescent intensity using Image Pro Plus software. Expression of each indicated protein in control mice was set to 100% (*n* = 6 images assessed from 3 mice per group) (left). Representative confocal images were shown (right). Indicated proteins were stained with appropriate primary antibodies plus Alexa Fluor-488-conjugated secondary antibody (green). Nuclei were counterstained with DAPI (blue). Scale bar, 25 μm. RhoB *P* = 0.0087, Beclin 1 *P* = 0.0022, LC3 *P* = 0.0022. **b** Western blotting analysis of indicated proteins in CFT073-infected mice. *n* = 3 mice. **c** Protein level of RhoB, Beclin 1 and LC3 in *RhoB*^+/−^ and *RhoB*^−/−^ mice infected by CFT073 through immunohistofluorescence analysis. Quantification of indicated proteins was shown, and expression of each indicated protein in WT mice was set to 100% (*n* = 6 images assessed from 3 mice per group) (left). Representative confocal images were shown (right). Scale bar, 25 μm. RhoB: WT vs. *RhoB*^+/−^ *P* < 1.0e−15, WT vs. *RhoB*^−/−^ *P* < 1.0e−15; Beclin 1: WT vs. *RhoB*^+/−^ *P* < 1.0e−15, WT vs. *RhoB*^−/−^ *P* < 1.0e−15; LC3: WT vs. *RhoB*^+/−^ *P* < 1.0e−15, WT vs. *RhoB*^−/−^ *P* < 1.0e−15. **d** Western blotting analysis of indicated proteins in CFT073-infected *RhoB*^−/−^ mice. *n* = 3 mice. **e** Bacterial titers in bladders of WT, *RhoB*^+/−^ and *RhoB*^−/−^ mice inf**e**cted by CFT073 (*n* = 5 mice per group). WT vs. *RhoB*^+/−^ *P* = 0.0492, WT vs. *RhoB*^−/−^ *P* < 1.0e−15. **f** Schematic diagram illustrating RhoB-induced UPEC clearance. During UPEC in**f**ections, RhoB is rapidly induced upon LPS stimulation through TLR4-mediated signaling, possibly through the activated NF-κB pathway. RhoB forms a complex with Beclin 1 and Hsp90, which leads to increased stability of Beclin 1. As a result, more autophagosomes are formed, resulting in enhanced clearance of intracellular UPEC. Data are the mean ± SD, *P < 0.05, **P < 0.01, ***P < 0.001, ****P < 0.0001, two-tailed unpaired Mann−Whitney test (**a**), two-way ANOVA (**c**), or one-way ANOVA (**e**).

To determine the effects of UPEC infection on RhoB and autophagic proteins in vivo, we performed immunohistofluorescence (IHF) staining using bladder tissue sections from CFT073-infected mice as a model of acute UTI. Wild-type female C57BL/6J mice were transurethrally injected with 10^8^ CFU of CFT073 for 12 h before analysis. Stronger staining intensity of RhoB was detected at the urothelium beside the bladder lumen of infected mice, compared with that of control mice, as was higher staining intensity of LC3 and Beclin 1 (Fig. 6a). These coordinate enrichments of RhoB and autophagic proteins were also identified in bladder homogenates from infected mice using western blotting (Fig. 6b).

*RhoB* heterozygous (*RhoB*^+/−^) and knockout (*RhoB*^−/−^) mice were next employed in a model of acute UTI. Both IHF staining and western blotting assays indicated that the protein levels of RhoB, Beclin 1, and LC3 at 12 hpi in *RhoB*^+/−^ and *RhoB*^−/−^ mice were reduced compared with those of the wild-type mice (Fig. 6c, d and Supplementary Fig. 7a). As a result, bacterial titers in bladder homogenate from *RhoB*^+/−^ and *RhoB*^−/−^ mice infected by CFT073 were strongly increased compared with that from wild-type mice (Fig. 6e). However, under normal conditions, no significant physiological changes were observed in bladders of *RhoB*^+/−^ and *RhoB*^−/−^ mice, compared with that of wild-type mice based on H&E staining (Supplementary Fig. 7b).

Collectively, we conclude that UPEC induces RhoB level, which modulates Beclin 1 degradation and LC3 lipidation to promote intracellular UPEC clearance through the autophagy-related pathway (Fig. 6f).

## Discussion

The most studied autophagy protein involved in UPEC infection is ATG16L1, which is reported to enhance UPEC infection^9^. However, the mechanism through which ATG16L1 deficiency decreases UPEC infection is independent of autophagy but depends on superficial urothelial cell architectural alterations and enhanced IL-1β-mediated hyperinflammatory response in macrophages^10–13^. On the other hand, Atg16L1 is required to protect against *S. Typhimurium* infection through autophagy^28,36^. We examined the role of ATG16L1 in RhoB-mediated LC3 lipidation and UPEC clearance, and found that *RhoB* knockdown had no effect on ATG16L1 protein level with or without CFT073 infection, and vice versa (Supplementary Fig. 7c and d). Notably, *RhoB* knockdown-induced increase of intracellular UPEC is not restored by *ATG16L1* knockdown (Supplementary Fig. 7e). These results indicate that RhoB-mediated UPEC clearance may have no direct relationship with ATG16L1. Of note, a recent study from Xu et al. found that virulence factor SopF of *Salmonella* counteracted ATG16L1-mediated autophagy, which promoted intracellular bacterial survival^28^. It is possible that UPEC (generically similar to *Salmonella*) could also inhibit ATG16L1-mediated autophagy, as we observed that *ATG16L1* knockdown did not affect intracellular UPEC in vitro (Supplementary Fig. 7e), and similar results were also shown in previous studies^37,38^. In addition, as ATG16L1 mutation influence urothelial cell architecture to decrease intracellular UPEC, its effect on UPEC clearance in vitro may be affected by many factors. However, our work suggests that RhoB-mediated clearance of CFT073 is through the autophagy-related pathway, as the effects are inhibited by *Atg7* and *P62* knockdown, and RhoB promotes colocalization of UPEC with LC3. Thus, the effect of RhoB on UPEC clearance could be achieved through other autophagic mechanisms rather than ATG16L1 related pathways, which is complicated and need to be further studied.

It is also reported that ATG3 deficiency in mouse bladder superficial epithelium significantly increases UPEC bacterial load, and an autophagy-inducing peptide markedly reduces the bacterial burden of infected mouse bladders, which supports that autophagy enhances UPEC clearance^14^. In addition, mice with urothelial-cell-specific knockout of ATG16L1 (involved in phagophore elongation), ATG7 (involved in phagophore elongation), ATG14 (involved in phagophore initiation) or Epg5 (involved in autophagosome-lysosome fusion) have no effect on bacterial load in urine at an early stage (24 h post infection), while ATG16L1 and ATG7 (but not Atg14 and Epg5) deficient mice harbor fewer UPEC QIRs (quiescent intracellular reservoirs) in the bladder at 14 days post infection^13^. Taken together, different results of UPEC clearance in autophagy-deficient mice may be due to other effects except for autophagy in gene-deficient mice.

RhoB is a member of Rho GTPases family, which shares a high identity with RhoA and RhoC. Unlike constitutively expressed *RhoA* and *RhoC*, *RhoB* maintains low expression in resting cells and is upregulated to exhibit distinct cellular functions upon stimulations^18,39^. It has been demonstrated that RhoB can be induced in cells treated with LPS^19,21^ or bacterial toxins;^40^ however, the possible role of RhoB in bacterial infections is still unclear. Like most Rho GTPases, RhoB activity is mediated by recycling of GTP/GDP loading to regulate the dynamics of cytoskeleton organization^41^, inflammation^19,21^, and cancer progression^39^. RhoB differs from RhoA and RhoC mostly at the hypervariable region of its C-terminus, which is highly posttranslational modified and crucial for its subcellular localization^30^. Intriguingly, LC3 lipidation enhanced by RhoB is not due to its enzymatic function as Rho GTPases, but correlates with its posttranslational modification that determines its membrane-associated localization.

It has been shown that binding partners of Beclin 1 control its level by manipulating its ubiquitination^24^. Here, we showed RhoB enhanced Beclin 1 stability in an Hsp90 but not ubiquitination-dependent fashion. Intriguingly, RhoB also seemed to be a client protein of Hsp90 to maintain RhoB homeostasis. RhoB binds to Hsp90 through residues of 42–89, different from RhoB-Beclin 1 interaction, which supports the formation of RhoB-Beclin 1-Hsp90 complex. Obvious colocalization of RhoB and Beclin 1 was observed, and less colocalization of RhoB and CFT073 was detected, and *RhoB* overexpression has no effect on UPEC uptake or P62/Ubiquitin recruited to UPEC, which suggests that RhoB has not involved in bacteria phagocytosis and intracellular recognition. Taken together, RhoB has mainly involved in Beclin 1 stabilization, resulting in enhanced membrane nucleation, and may leave autophagosome and was recycled during the late phase of autophagosome including bacteria, which should be further studied.

S-guanylated Group A Streptocccus by 8-nitro-cGMP, induced by nitric oxide, is reported to be selected for autophagic degradation^42^. We found that suppression of 8-nitro-cGMP by NaHS, or a nitric oxide synthase inhibitor L-NMMA, did not significantly affect RhoB effect on intracellular CFT073 survival, indicating that S-guanylation is not related to RhoB-mediated UPEC clearance (Supplementary Fig. 7f and g).

In summary, we identified the interaction between RhoB-Beclin-1-Hsp90 that promoted LC3 lipidation and intracellular UPEC clearance, providing the potential target to develop treatment strategies for UTIs. Whether RhoB has an effect on the clearance of other intracellular bacteria through the same mechanism should be examined in further studies.

## Methods

### Cell lines and reagents

Human kidney epithelial HEK293 cells (ATCC CRL-1573), which is used for transfection experiments in this study, were grown in Dulbecco’s modified Eagle’s medium (DMEM) basic, supplemented with 10% fetal bovine serum (FBS) and/or 100 units/ml penicillin/streptomycin. Human bladder epithelial 5637 cells (ATCC HTB-9, a commonly used bladder epithelial cell line), J82 cells (ATCC HTB-1) and T24 cells (ATCC HTB-4) as well as human prostate epithelial PC-3 cells (ATCC CRL-1435) were maintained in RPMI1640 and 10% FBS. All cell lines were routinely cultured at 37 °C in a humidified incubator containing 5% CO_2_.

The proteasome inhibitor MG-132 (M7449), 3×FLAG Peptide (F4799), c-Myc Peptide (M2435), Lipopolysaccharides from *Escherichia coli* O55:B5 (L6529), L-Glutathione reduced (G4251), and Sodium hydrosulfide (161527) were purchased from Sigma (St. Louis, MO, USA). The proteasome inhibitor bafilomycin A_1_ (BafA1, ab120497) was purchased from Abcam (Cambridge, UK). Gentamycin sulfate (B540724) was purchased from Sangon Biotech (Shanghai, China). Rhotekin-RBD beads (RT02A) and Rhodamine Phalloidin (PHDR1) were purchased from Cytoskeleton (Denver, CO, USA). Geldanamycin (GA, HY-15230), PEG300 (HY-Y0873), L-NMMA (HY-18732A), and cycloheximide (CHX, HY-12320) was purchased from MedChem Express (Monmouth Junction, NJ, USA). Tween 80 (CSN21336) were purchased from CSNpharm (Shanghai, China).

### DNA constructs

Human RhoB was amplified by PCR and cloned to pCMV-tag2b vector with an N-terminal FLAG tag (FLAG-RhoB), or cloned to p3XFLAG-CMV-10 vector with an N-terminal 3XFLAG tag (3XFLAG-RhoB). Human Beclin 1 and Hsp90 were cloned to pCDNA 3.1 (-) vector with MYC tags at both N- and C-terminus (MYC-Beclin 1) or a HA tag at N-terminus (HA-Hsp90), respectively. The single mutants of RhoB (T19N, Q63E, Q63L, S185A, C193S, T129V, R133A, and Q136G), were generated by a round circle polymerase chain reaction-based site-directed Fast Mutagenesis System kit (TransGen Biotech, Beijing, China) according to the manufacturer’s protocol. Truncation mutants of RhoB and Beclin 1 were amplified by PCR and cloned to pCDNA 3.1 (-) vector with an N-terminal 3×FLAG or MYC tags at both N- and C-terminus, respectively. The mCherry-GFP-LC3, mCherry-LC3, and GFP-LC3 construct were purchased from MiaoLing Plasmid Sharing Platform (Miaolingbio, Wuhan, China). The constructed plasmids and primers are listed in Supplementary Data 1 and 2.

### Bacterial strains and invasion in cultured cells

*Escherichia coli* strain CFT073 was grown in Luria-Bertani (LB) medium overnight at 37 °C. For bacterial invasion assay, CFT073 were subcultured (1:100) into fresh LB and inoculated for 2 h at 37 °C. The log-phase grown bacteria were then subjected to invasion of 5637 bladder epithelial cells in 12-well plates at indicated MOI. To enhance the efficiency of bacteria invasion, we applied a short spinoculation at 500 × *g* for 5 min after the addition of bacteria. After that, infected cells were additionally inoculated for 30 min at 37 °C followed by treatment of 100 μg/ml gentamycin for 2 h to kill extracellular bacteria. Infected cells were then washed thoroughly with PBS to remove the bacteria remaining in supernatants. Lysates containing intracellular bacteria were next extracted by 0.2% TritonX-100 and plated on LB agar overnight before colony-forming units (CFU) were assessed. For bacterial entry assay, infected cells were treated with gentamycin for 30 min to kill extracellular bacteria.

For bacterial expulsion assay, infected cells were treated with 100 ug/ml gentamycin plus 100 mM methyl α-D-mannopyranoside (Sigma) for 1 h to prevent reattachment and entry of bacteria. Infected cells were thus washed three times with PBS and treated with culture media containing 100 mM methyl α-D-mannopyranoside, 25 μg/mL trimethoprim (TMP), and 125 μg/mL sulfamethoxazole (SMZ) for 4 h to prevent bacterial growth. Culture media from infected cells was thus collected and plated on LB agar plates for assessment of CFU.

### Antibodies and western blotting

Anti-RhoB (C-5, sc-8048, 1:500 for western blotting), Alexa Fluor 488-conjugated anti-RhoB (sc-8048 AF488, 1:200 for fluorescent imaging) and anti-TLR4 (sc-293072, 1:500 for western blotting) antibodies were purchased from Santa Cruz Biotechnology (Dallas, TX, USA). Anti-β-actin (A1978, 1:10,000 for western blotting), anti-FLAG (F3165, 1:5000 for western blotting), and anti-LC3B (L7543, 1:10000 for western blotting, 1:100–500 for fluorescent imaging) antibodies were purchased from Sigma-Aldrich (St. Louis, MO, USA). Anti-ATG5 (9980, 1:1000 for western blotting), anti-ATG7 (8558, 1:1000 for western blotting), anti-Beclin 1 (3495 and 4122, 1:1000 for western blotting, 1:100–500 for fluorescent imaging), anti-HA (2367 and 3724, 1:1000 for western blotting), anti-Hsp90 (8165 s, 1:1000 for western blotting), anti-Rubicon (8465, 1:1000 for western blotting), anti-SQSTM1/P62 (8025 and 7695, 1:1000 for western blotting, 1:100 for fluorescent imaging) and anti-ATG16L1 (D6D5, 1:1000 for western blotting) antibodies were purchased from Cell Signaling Technology (Danvers, MA, USA). Anti-LPS (ab35654, 1:100 for fluorescent imaging) antibody was purchased from Abcam (Cambridge, UK,). Anti-Myc (1A5A2, 1:1000 for western blotting), CoraLite 594 conjugated Goat anti-mouse (SA00013-3, 1:100 for fluorescent imaging) and Goat anti-rabbit (SA00013-4), AMCA-conjugated Goat anti-mouse (SA00010-1, 1:100 for fluorescent imaging) and Goat anti-rabbit (SA00010-2, 1:100 for fluorescent imaging) antibodies were purchased from Proteintech (Rosemont, IL, USA). Anti-ubiquitinylated proteins (FK2, 1:200 for fluorescent imaging) antibody was purchased from Millipore (now Sigma-Aldrich). Alexa Fluor 405-conjugated goat anti-rabbit antibody (A31556, 1:100 for fluorescent imaging) was purchased from Invitrogen (Eugene, OR, USA). Cells were lysed in RIPA lysis buffer (R0020, Solarbio, Beijing, China) with the addition of protease inhibitors (Roche, Basel, Switzerland). Whole-cell lysates were then clarified by centrifugation and boiled in SDS loading buffer, followed by 12% or 15% SDS electrophoresis. Immunoblotting was performed with indicated primary antibodies and corresponding HRP-conjugated secondary antibodies. Signals were detected by Amersham Imager 600 with integrated analysis software (GE Healthcare Life Sciences, Buckinghamshire, United Kingdom) and analyzed using NIH ImageJ software (National Institutes of Health, Bethesda, USA).

### Immunoprecipitation assays

Cells were washed with pre-chilled PBS and lysed in lysis buffer (0.2 mM EDTA, 50 mM Tris-HCl (pH7.4), 150 mM NaCl, 1 % NP-40) containing freshly added protease inhibitor (Roche) on ice. Cell lysates were centrifuged to remove cell debris. For immunoprecipitation of FLAG- or MYC-tagged proteins, cell lysates were incubated with either anti-FLAG beads (A2220, Sigma) or anti-c-MYC beads (A7470, Sigma) in lysis buffer overnight at 4 °C with constant rotation, followed by centrifugation at 500 × *g* and washing with binding buffer (2 mM EDTA, 50 mM Tris-HCl (pH 7.4), 150 mM NaCl, 0.1% NP-40, 30% glycerol). Beads were then incubated with corresponding 3×FLAG or c-MYC peptides (Sigma) in TBS buffer (20 mM Tris-HCl, 140 mM NaCl, PH 7.6) on ice for 2 h and centrifuged again to collect the supernatants. For immunoprecipitation of endogenous proteins, cells were treated by LPS for 2 h if needed. Cell lysates were then prepared and incubated with anti-RhoB (Santa cruz), anti-Beclin 1 (CST) or immunoglobin G (IgG) (CST, 3420 S) overnight at 4 °C, and subsequently incubated with Protein A/G beads (Thermo Fisher, 20241) for 2 h. Beads were thoroughly washed with binding buffer, boiled in SDS loading buffer, and subjected to SDS-PAGE and western blotting.

For detecting ubiquitination of Beclin 1, plasmids encoding HA-tagged ubiquitin with substitutions of all lysine residues by arginine except K11 (HA-Ub K11) or K48 (HA-Ub K48) were co-transfected into HEK293 cells together with other plasmids encoding FLAG-RhoB and MYC-Beclin 1. Transfected cells were treated with or without GA (1 μM) and MG-132 (10 μM) overnight. Immunoprecipitation of MYC-Beclin 1 was carried out as above described followed by western blotting analysis with anti-HA antibody and appropriate secondary antibody.

### Recombinant protein purification and Glutathione S-transferase (GST) pull-down assay

For recombinant protein expression, pGEX4T-3 vector was used to generate constructs encoding GST-fused proteins, including RhoB, Beclin 1, Hsp90 and related mutants. These GST-fused proteins were expressed overnight in BL21 (DE3) with 100 μg/ml ampicillin followed by induction of 100 μM IPTG at 16 °C. Cells were then pelleted by centrifugation at 10,000 × *g* and resuspended into lysis buffer (50 mM Tris, 100 mM NaCl, 1 mg/ml lysozyme, 1% PMSF, PH 7.9) followed by sonication for 60 cycles. The mixture was then treated with lysis buffer (10 mM MgCl_2_, nuclease, 1% Triton) and centrifuged again to remove cell debris. Purification and elution of GST-fused proteins were performed by using Glutathione Sepharose 4B (GE Healthscience) according to the manufacturer’s instruction. Protein concentration was tested using the BCA Protein Assay Kit (Thermo Fisher) and confirmed by western blotting with specific antibodies.

For GST pull-down assays, ~1 μg of each GST-fused protein indicated was combined in binding buffer (0.8% BSA in PBS containing freshly added PMSF) at 4 °C overnight. The mixture was next incubated with anti-RhoB or anti-Beclin 1 antibodies (IgG served as negative control) plus protein G Sepharose overnight at 4 °C with constant rotation. Beads were then centrifuged and washed five times with binding buffer, resolved, and boiled in SDS sample buffer. Samples were then subjected to western blotting with anti-RhoB (Santa Cruz), anti-Beclin 1 (CST) or anti-Hsp90 antibody (CST).

### Immunofluorescence staining of cells

Cells were pre-cultured in LAB-TEK 4-well chamber slides, vigorously washed with sterilized PBS, and fixed with 4% paraformaldehyde at room temperature. After that, fixed cells were permeablized with 0.5% Triton X-100/PBS for 20 min at room temperature, followed by blocking with 5% BSA/PBS containing 10% (v/v) goat serum. Cells were stained by specific primary antibodies plus appropriate Alexa Fluor 488/CoraLite 594-conjugated secondary antibody. If needed, F-actin was labeled with 100 nM Rhodamine Phalloidin in PBS, and nuclei were counterstained with DAPI. Images were taken using a confocal fluorescence microscope (Leica TCS-SP8, Leica Microsystems, Germany).

### Transmission electron microscopy (TEM)

TEM analysis of cultured cells was performed according to the previously described method^43^. Briefly, cells were trypsinized, washed with pure FBS, and then fixed with PBS containing 3% glutaraldehyde and 2% paraformaldehyde at 4 °C overnight. After that, fixed cells were washed three times with PBS and post-fixed in 1% osmium tetroxide at room temperature for 45 min, and then stained with 1% uranyl acetate. Samples were subsequently dehydrated in a graded series of ethanol and embedded in Embed 812 resin, and were polymerized at 70 °C for 2 days. Ultrathin sections were cut, mounted, and stained with uranyl acetate and lead citrate. Images were acquired using a TEM (Hitachi, HT7700, Tokyo, Japan). Numbers of autophagic vacuoles were manually counted.

### GFP-LC3 transient transfection

Cells were co-transfected with plasmids encoding mCherry-EGFP-LC3B or GFP-LC3 in the presence of RhoB wild-type (WT) and mutants using PEI following the manufacturer’s instruction. At 48 h post transfection, cells were treated with LPS if needed. The numbers of mCherry positive (Red), GFP positive (Green), and both positive (Yellow) puncta in each cell were manually counted using a confocal fluorescence microscopy (Leica TCS SP8; Leica Microsystems).

For detecting GFP-LC3 decorated bacteria, 5637 bladder epithelial cells were seeded in LAB-TEK 4-well chamber slides and transfected with GFP-LC3 for 48 h. Cells were then infected by CFT073 at MOI of 100 for 30 min followed by washing and treatment of 100 μg/ml gentamycin for 1 h. Infected cells were thoroughly washed with PBS, fixed and permeablized. Anti-LPS antibody and CoraLite 594-conjugated secondary antibody were used to detect intracellular bacteria. Images were acquired by Leica confocal fluorescence microscopy (Leica Microsystems).

For detecting colocalization of GFP-CFT073 with P62, Ub, and LC3 in 5637 cells, mCherry-LC3, RhoB, or Vector were transfected into 5637 cells in LAB-TEK 4-well chamber slides for 48 h. Cells were then infected with GFP-CFT073 for 1 h at MOI of 50. Infected cells were then washed, fixed, and permeablized, followed by immunostaining with anti-P62 or anti-Ub antibodies plus AMCA-conjugated secondary antibodies.

### Measurement of RhoB activation

To determine the activation state of each RhoB construct, the Activation Assay Biochem Kits containing Rhotekin-RBD beads **(**Cytoskeleton**)** were used. HEK293 cells were transfected with FLAG-tagged RhoB-WT and mutants for 48 h. Cellular extracts were then prepared and subjected to IP with Rhotekin-RBD beads according to the manufacturer’s instruction. Western blotting with anti-RhoB antibody was performed and the amount of activated RhoB (GTP-bound) was thereby assessed.

### RNA interference

siRNAs for indicated genes were synthesized and tested along with a scrambled siRNA control (siScr) (GenePharma, Shanghai, China). Appropriate amounts of siRNAs were transfected using Lipofectamine 3000 (Invitrogen) according to the manufacturer’s instruction. At 48 h post transfections, cellular extracts were prepared and subjected to western blotting with corresponding antibodies. Clones with the highest efficiency were used in the following experiments. Sequences of these siRNAs are listed in Supplementary Data 2.

### RNA extraction and qRT-PCR

Total RNA was extracted using a Total RNA Extraction Kit (Solarbio, Beijing, China) in accordance with the manufacturer’s instructions. cDNA of target protein was then generated using a Revert Aid First Strand cDNA Synthesis Kit (Thermo Scientific) with specific primers listed in Supplementary Data 2. FastStart Universal SYBR Green Master Mix (Roche) was used in PCR reactions. The PCR reactions were set as follows: preincubation (95 °C for 5 min), 45 cycles of 3-step amplification (95 °C for 20 s, 60 °C for 20 s, 72 °C for 20 s, and 78 °C for 1 s), melting (95 °C for 10 s, 65 °C for 60 s, 97 °C for 1 s), and cooling (37 °C for 30 s). The transcription level of GAPDH served as the endogenous control. All data were normalized and calculated via the comparative critical threshold cycle 2^−∆∆Ct^ method.

### Animal model of acute UTI

RhoB heterozygous (*RhoB*^+/−^) and knockout (*RhoB*^−/−^) mice were generated at the Shanghai Biomodel Organisms Center and were confirmed by PCR genotype analysis. Female C57BL/6J mice, aged 6–8 weeks, were purchased from the Academy of Military Medical Science (Beijing, China). Wild-type (C57BL/6J), heterozygous (*RhoB*^+/−^), and knockout (*RhoB*^−/−^) six- to eight-week-old female mice were used in all animal studies according to the Guide for the Care and Use of Laboratory Animals. All mice were maintained in the conditions as follows: 12-h light/12-h dark cycle, 24 °C, 30–60% humidity. Approvals for the use of these mice were obtained from the Animal Ethics Committee at Tianjin Medical University.

In the model of acute UTI, UPEC strain *E. coli* CFT073 was pre-cultured overnight in LB medium at 37 °C, harvested by centrifugation, and resuspended in sterilized PBS. For bacterial infection, about 10^8^ CFU of CFT073 were transurethrally injected into mice after anesthesia. After 12 h incubation, infected mice were sacrificed by cervical dislocation, and the bladders were aseptically excised and homogenized in 0.025% Triton X-100/PBS. CFU were next determined using appropriate amount of bladder homogenate by serial dilution. For detecting the level of autophagic proteins indicated, homogenate of bladders from infected mice were alternatively prepared in RIPA lysis buffer, boiled in SDS sample buffer and subjected to western blotting.

### Immunofluorescence staining of tissues

Frozen sections of bladders from infected mice were prepared as previously reported^44^. In brief, bladders from infected mice were aseptically harvested and embedded in OCT compound with liquid nitrogen. Frozen sections of 5 μm were carefully cut and placed on glass slides to allow them air-dried at room temperature. After that, sections were fixed in pre-chilled acetone and submerged in pure methanol and methanol containing 3% hydrogen peroxide. Sections were then rehydrated in PBS, blocked in 5% BSA/PBS and incubated with indicated primary antibodies overnight at 4 °C. After washing with cold PBS, sections were stained using Alexa Fluor 488-conjugated secondary antibodies and DAPI. Images were taken using Leica TCS SP8 microscope (Leica Microsystems). Densitometric analysis of indicated protein was performed using Image Pro Plus software (Media Cybernetics, Rockville, USA).

### H&E staining

Fixed bladders from mice were embedded in paraffin. After that, tissue sections of 5 μm were made and subjected to hematoxylin and eosin (H&E) staining. Images were acquired using Olympus microscope (Tokyo, Japan).

### Statistical analysis

Data were presented as the mean ± SD. Statistical significance of the differences between groups was calculated using Student’s *t* test or ANOVA analysis. To analyze the bacterial titers in the UTI mouse models, the non-parametric Mann–Whitney test or ANOVA were used to calculate the statistical significance.

### Reporting summary

Further information on research design is available in the Nature Research Reporting Summary linked to this article.

## Supplementary information

Supplementary Figures

Supplementary Data 1

Supplementary Data 2

Description of Additional Supplementary Files

Reporting Summary

## Data Availability

Source data are provided with this paper. The structural data of RhoB are available in the Protein Data Bank with PDB identifier 2FV8. All the data that support the findings and unique materials in this study are available from the corresponding author [Q.W.] or the first author [C.M.] on reasonable request through an MTA. Source data are provided with this paper.

## Source data

Source Data
