# Supplementary material for: An infection-induced RhoB-Beclin 1-Hsp90 complex enhances clearance of uropathogenic Escherichia coli
Source: Nat Commun. 2021 May 10;12:2587. doi: 10.1038/s41467-021-22726-8 (PMC8110956; doi:10.1038/s41467-021-22726-8)
Supplement: Supplementary file 1 — Supplementary Figures [file 41467_2021_22726_MOESM1_ESM.pdf]

An infection-induced RhoB-Beclin 1-Hsp90 complex enhances clearance of uropathogenic *Escherichia coli*

Chunhui Miao[a,1], Mingyu Yu[a,1], Geng Pei[a], Zhenyi Ma[b], Lisong Zhang[c], Jianming Yang[a], Junqiang Lv[a], Zhi-Song Zhang[c], Evan T. Keller[d], Zhi Yao[a,e,*], Quan Wang[a,*]

[a]Department of Immunology, Key Laboratory of Immune Microenvironment and Disease of the Educational Ministry of China, Tianjin Key Laboratory of Cellular and Molecular Immunology, School of Basic Medical Sciences, Tianjin Medical University, Tianjin 300070, China.

[b]Department of Biochemistry and Molecular Biology, School of Basic Medical Sciences, Tianjin Key Laboratory of Medical Epigenetics, Tianjin Medical University, China.

[c]State Key Laboratory of Medicinal Chemical Biology and College of Pharmacy, Collaborative Innovation Center for Biotherapy, Tianjin Key Laboratory of Molecular Drug Research, Nankai University, Tianjin 300350, China.

[d]Departments of Urology, University of Michigan, Ann Arbor, Michigan, USA.

[e]2011 Collaborative Innovation Center of Tianjin for Medical Epigenetics, Tianjin Medical University, Tianjin 300070, China.

[1]These authors contributed equally to this study

***Corresponding Author:** Quan Wang, Department of Immunology, Tianjin Medical University, 22 Qixiangtai Road, Heping District 300070, Tianjin, China; Tel.: 86-22-83336817; Fax: 86-22-83336811; E-mail address: wangquan@tmu.edu.cn; or Zhi Yao, Department of Immunology, Tianjin Medical University, 22 Qixiangtai Road, Heping District 300070, Tianjin, China; Tel.: 86-22-83336817; Fax: 86-22-83336811; E-mail address: yaozhi@tmu.edu.cn.

The authors declare no conflict of interests.

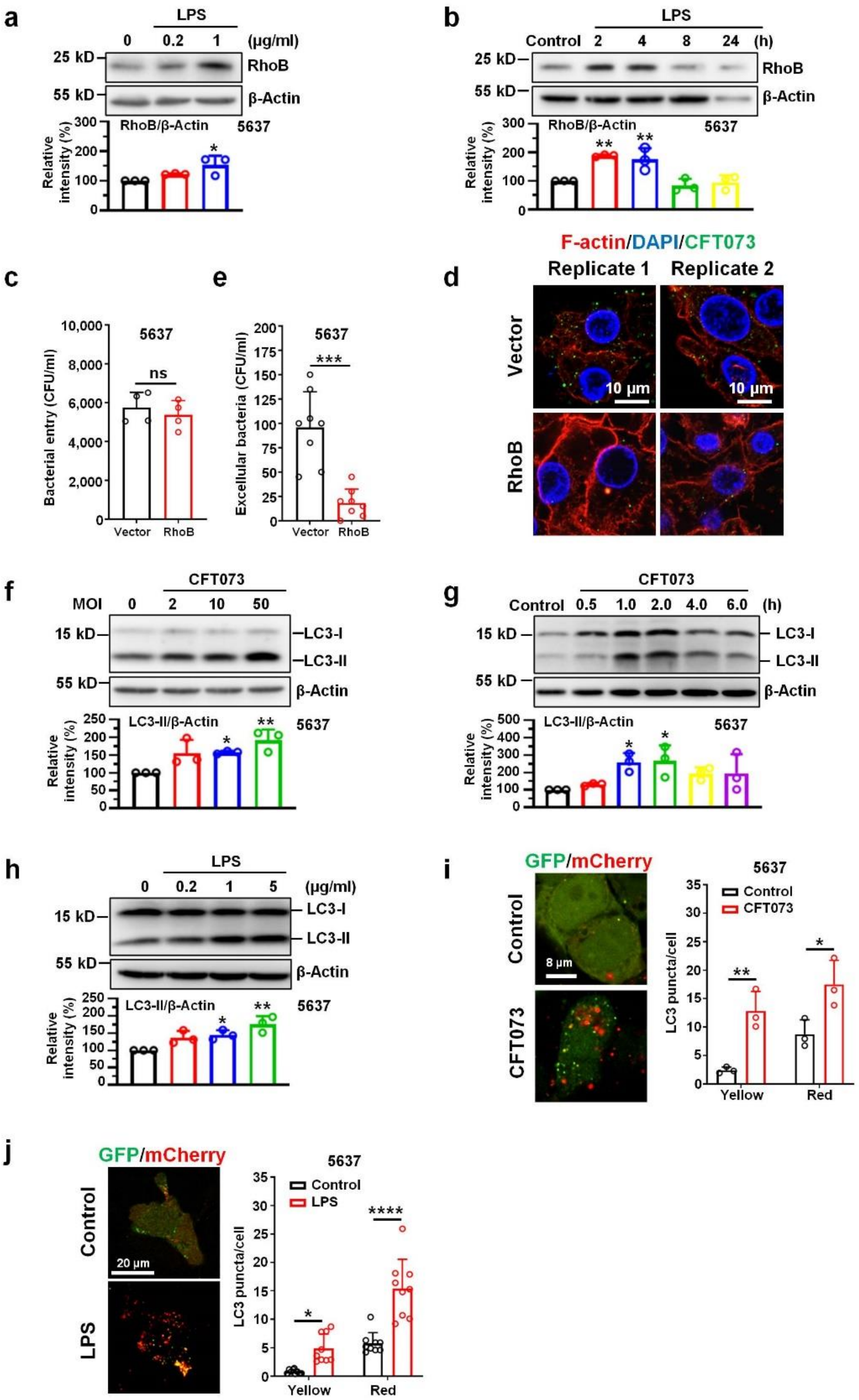

Supplementary Figure 1. Bacterial infection induces RhoB level and LC3 lipidation. a-b, RhoB level in 5637 cells with treatment of LPS. 5×10^4 of 5637 cells per well were seeded in 24-well plate and treated with LPS at indicated concentrations for 2 h (**a**) or treated with 1 ug/mL LPS for indicated periods of time (**b**). The RhoB density was normalized to that of β-Actin. The relative density of untreated cells was set to 100%. n = 3 independent experiments. (**a**) RhoB/β-Actin: 0 vs. 0.2 $P = 0.2892$, 0 vs. 1 $P = 0.0204$. (**b**) RhoB/β-Actin: 0 vs. 2 $P = 0.0028$, 0 vs. 4 $P = 0.0083$, 0 vs. 8 $P = 0.8200$, 0 vs. 24 $P = 0.9961$. **c**, Effect of RhoB on bacterial entry of CFT073 in 5637 cells. Infected cells were incubated in 37 °C for 30 min followed by washing and

treatments with gentamycin. At 30 min post gentamycin treatment, infected cells were lysed and CFU of intracellular bacteria were measured. *P* = 0.4857. n = 4 independent experiments. **d**, The representative images in replicated experiments of Fig. 1d were shown. Scale bar, 10 μm. Three independent experiments. **e**, Effect of RhoB on bacterial expulsion of CFT073 from 5637 cells. *RhoB*- or vector-transfected 5637 cells were infected by CFT073 at MOI of 50 followed by treatment of gentamycin and inhibitors as described in Method. At 4 hpi, the number of extracellular CFT073 in culture media was measured. n = 8 independent experiments. *P* = 0.0003. **f-h**, Autophagy is promoted in 5637 cells infected by CFT073 at indicated MOIs for 2 h (**f**), infected by CFT073 at MOI of 50 for indicated times (**g**), or treated by LPS with indicated concentrations for 2 h (**h**). The LC3-II density was normalized to that of β-Actin. The relative densities of untreated or uninfected cells were set to 100%. n = 3 independent experiments. (**f**) LC3-II/β-Actin: 0 vs. 2 *P* = 0.0525, 0 vs. 10 *P* = 0.0451, 0 vs. 50 *P* = 0.0038. (**g**) LC3-II/β-Actin: control vs. 0.5 *P* = 0.9619, control vs. 1 *P* = 0.0374, control vs. 2 *P* = 0.0278, control vs. 4 *P* = 0.2969, control vs. 6 *P* = 0.2831. (**h**) LC3-II/β-Actin: 0 vs. 0.2 *P* = 0.0595, 0 vs. 1 *P* = 0.0257, 0 vs. 5 *P* = 0.0012. **i-j**, Puncta formation of mCherry-GFP-LC3 in 5637 cells infected with CFT073 for 2 h (**i**), or treated with LPS for 2 h (**j**). The numbers of autophagosome and autolysosome were counted per cell. Scale bar, 8 μm or 20 μm. (**i**) n = 3 random areas assessed from 3 independent experiments. Yellow *P* = 0.0058, Red *P* = 0.0150. (**j**) n = 9 random areas assessed from 3 independent experiments. Yellow *P* = 0.0153, Red *P* = 2.8e-7. Data are the mean ± SD, **P* < 0.05, ***P* < 0.01, ****P* < 0.001, *****P* < 0.0001, two-tailed unpaired Student's *t* test (c, e), one-way ANOVA (a-b, f-h), or two-way ANOVA (i-j).

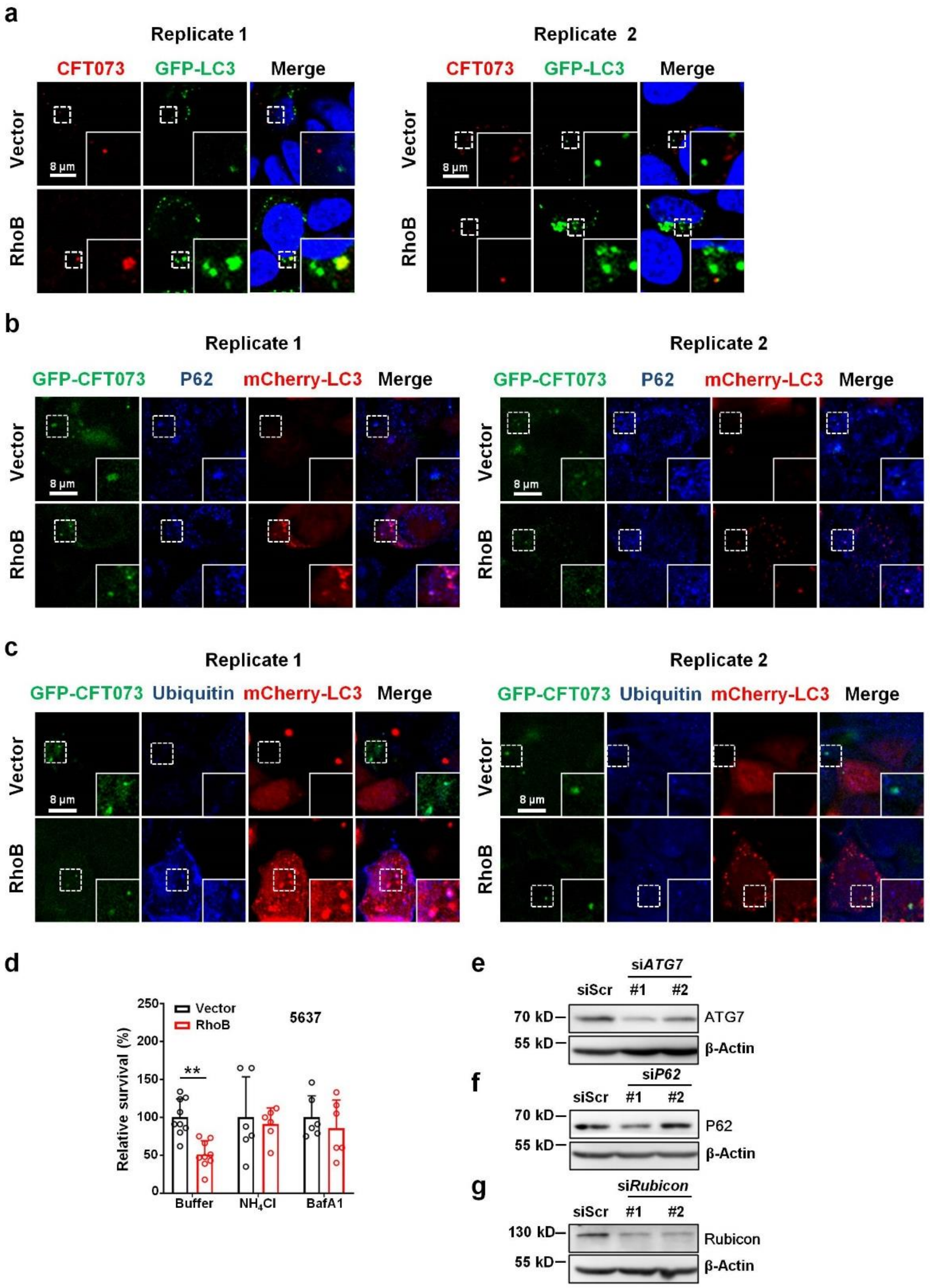

Supplementary Figure 2. RhoB enhances LC3-decoration of intracellular CFT073. **a-c**, The representative images in replicated experiments of Fig. 1f (**a**), g (**b**) and h (**c**) were shown. Scale bar, 8 μm. Three independent experiments. **d**, Bacterial survival in *RhoB*-overexpressing 5637 cells with treatments of NH_4Cl and BafA1. 5637 cells were transfected with *RhoB* or vector for 48 h. Cells were pre-treated with 30 mM NH_4Cl or 50 nM BafA1, followed by bacterial invasion assay. Bacterial survival of vector-transfected cells in each group was set to 100%. Buffer: n = 9 independent experiments, $P = 0.0057$; NH_4Cl and BafA1: n = 6 independent experiments, NH_4Cl $P = 0.9473$, BafA1 $P = 0.8140$. **e-g**, Knockdown efficiency of siRNAs for autophagy markers, ATG7 (**e**), P62 (**f**) and Rubicon (**g**) in 5637 cells. n = 3 independent experiments. Data are the mean ± SD, $^{**}P < 0.01$, two-way ANOVA (d).

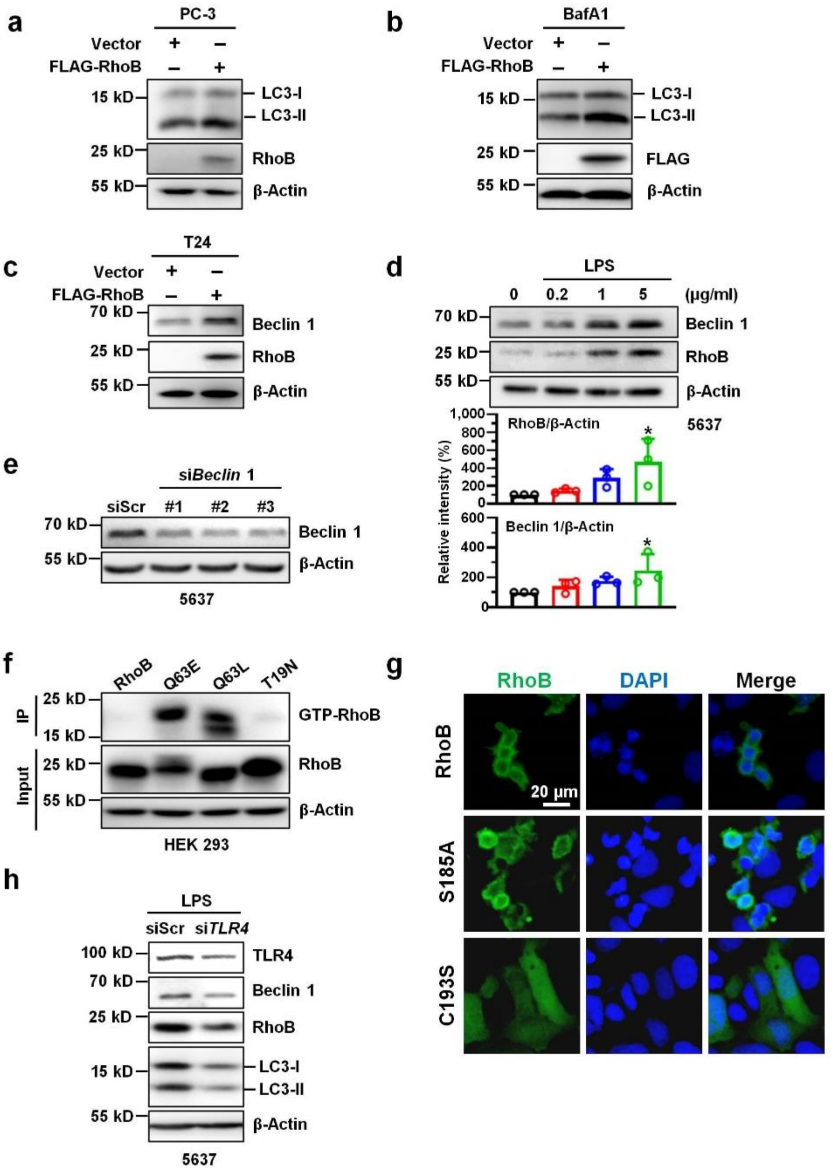

Supplementary Figure 3. RhoB promotes LC3 lipidation in correlation with its localization. **a**, LC3 lipidation in PC-3 cells transfected with *RhoB*. n = 3 independent experiments. **b**, LC3 lipidation in *RhoB*-overexpressing HEK293 cells in the presence of BafA1. n = 3 independent experiments. **c**, Effect of RhoB on endogenous Beclin 1 in T24 cells. n = 3 independent experiments. **d**, Protein level of endogenous RhoB and Beclin 1 in LPS-treated 5637 cells. The densities of RhoB and Beclin 1 were normalized to that of β-Actin. The relative density of untreated cells was set to 100%. Data are the mean ± SD. *P <0.05, one-way ANOVA. n = 3 independent experiments. RhoB/β-Actin: 0 vs. 0.2 $P = 0.9568$, 0 vs. 1 $P = 0.2959$, 0 vs. 5 $P = 0.0288$; Beclin 1/β-Actin: 0 vs. 0.2 $P = 0.7592$, 0 vs. 1 $P = 0.3554$, 0 vs. 5 $P = 0.0463$. **e**, Knockdown efficiency of *Beclin 1* siRNAs in 5637 cells. n = 3 independent experiments. **f**, Activation of RhoB constructs was analyzed. HEK 293 cells transfected with RhoB WT, Q63E, Q63L and T19N were lysed at 48 h post transfection and subjected to immunoprecipitation with Rhotekin-RBD beads. n = 3 independent experiments. **g**, Representative images showing localization of RhoB-WT, S185A and C193S stained by Alexa Fluor 488-conjugated anti-RhoB antibody. Scale bar, 20 μm. n = 3 independent experiments. **h**, Knockdown of *TLR4* decreases levels of RhoB and autophagic markers in LPS-treated 5637 cells. 5637 cells were transfected with *TLR4* siRNA for 48 h followed by treatment

of LPS for 2 h. Cells were then lysed and subjected to western blotting analysis. n = 3 independent experiments.

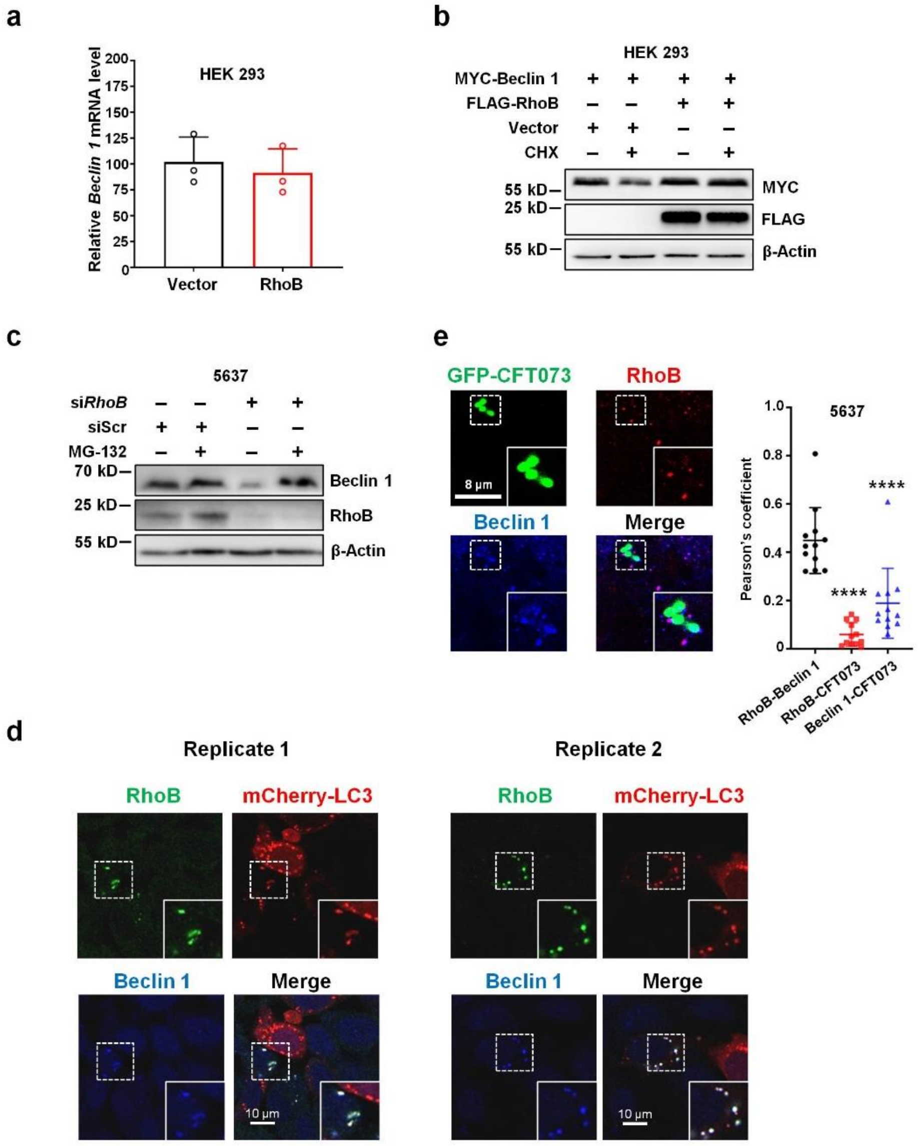

Supplementary Figure 4. RhoB affects the protein stability of Beclin 1. a, qRT-PCR analysis of *Beclin 1* mRNA from HEK 293 cells transfected with FLAG-RhoB or vector. Data are the mean ± SD, two-tailed unpaired Student's *t* test. n = 3 independent experiments. P = 0.7000. **b**, Protein level of Beclin 1 in *RhoB*-overexpressinbg HEK 293 cells in the presence or absence of cycloheximide (CHX). HEK 293 cells were transfected with plasmids encoding FLAG-RhoB or vector. At 36 h post transfection, cells were treated with CHX (25 µg/ml) overnight and lysed. n = 3 independent experiments. **c**, Protein level of endogenous Beclin 1 in *RhoB* knocked down 5637 cells in the presence or absence of MG-132. 5637 cells were transfected with *RhoB* siRNAs or siScr. At 48 h post transfection, cells were treated with 10 µM MG-132 for 6 h and then lysed. n = 3 independent experiments. **d,** The representative images in replicated experiments of Fig. 3g were shown. Scale bar, 10 µm. Three independent experiments. **e,** Colocalization of endogenous RhoB and Beclin 1 with GFP-CFT073 in 5637 cells (left). 5637 cells were infected with GFP-CFT073 for 2 h followed by immunostaining with anti-RhoB (red) and anti-Beclin 1 antibodies (blue). Quantification of

colocalization was shown by calculating the Pearson correlation coefficients (*R* values) using Image Pro Plus software (right). n = 10 random areas per group from three independent experiments. Scale bar, 8 μm. Pearson's coefficients for RhoB-Beclin 1, RhoB-CFT073 and Beclin 1-CFT073 colocalization were ~0.45, ~0.06 and ~0.19, respectively. RhoB-Beclin 1 vs. RhoB-CFT073 P = 5.3e-9, RhoB-Beclin 1 vs. Beclin 1-CFT073 P =1.2e-5. Data are the mean ± SD, ****P < 0.0001, one-way ANOVA.

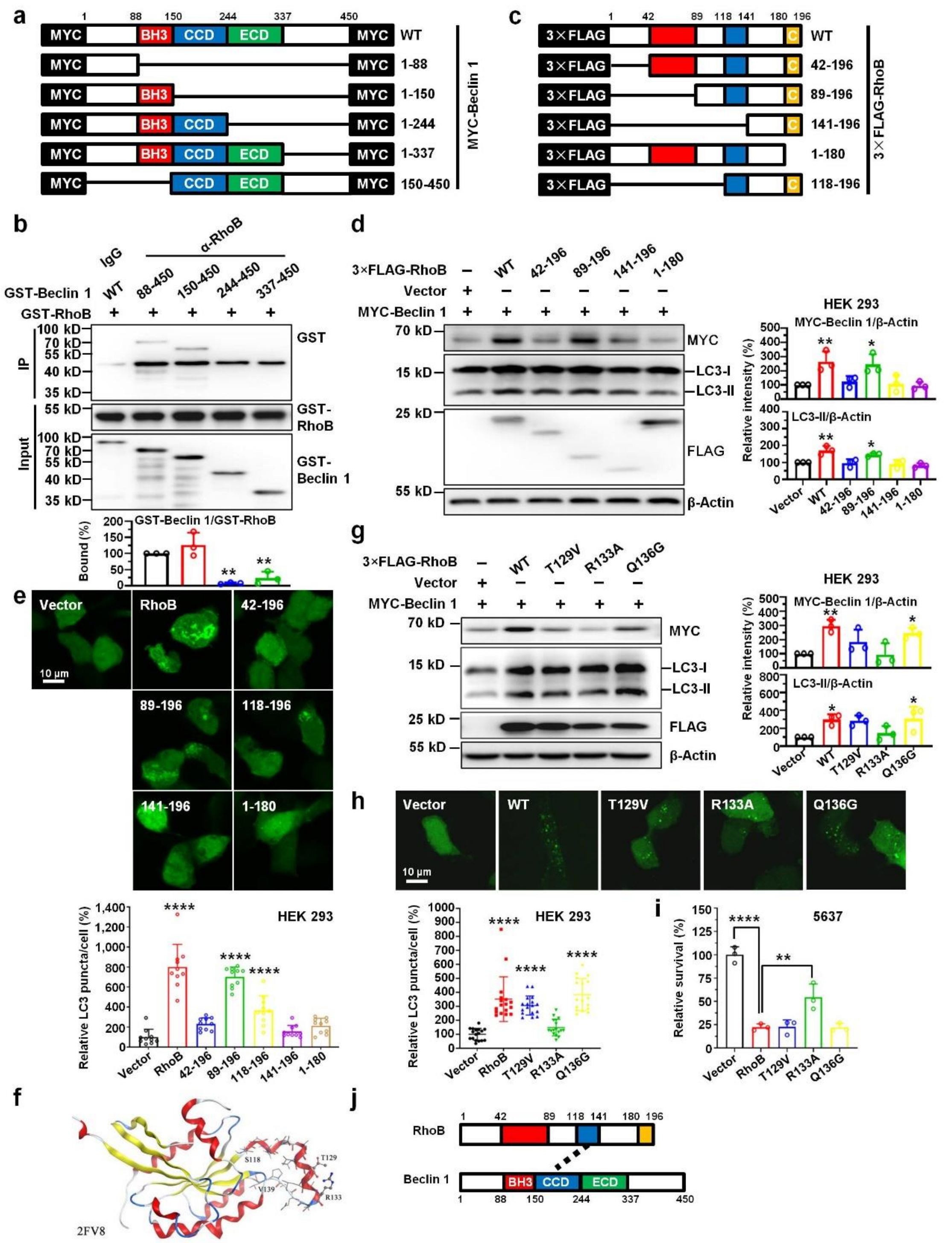

Supplementary Figure 5. Effects of RhoB-Beclin 1 interaction on Beclin 1 stabilization, LC3 lipidation and UPEC clearance. a, Schematic diagram of Beclin 1 and RhoB truncation mutants used in this study. MYC, MYC tag; BH3, Bcl-2-homology-3 domain; CCD, coil-coil domain; ECD, evolutionary conserved domain. **b**, GST pull-down assay with GST-fused RhoB and truncated GST-Beclin 1 proteins. Immunoprecipitation was performed with anti-RhoB antibody or IgG. The density of GST-Beclin 1 was normalized to that of GST-RhoB in IP group. The percentage of bound GST-Beclin1 88-450 to GST-RhoB was set to 100%. n = 3 independent experiments. GST-Beclin 1/GST-RhoB: 88-450 vs. 150-450 P = 0.3355, 88-450 vs. 244-450 P = 0.0018, 88-450 vs. 337-450 P = 0.0059. **c**, Schematic diagram of RhoB

truncation mutants used in this study. 3 × FLAG, 3 × FLAG tag; C, C-terminal hypervariable region. **d**, Effects of RhoB truncation mutants on LC3 lipidation. HEK293 cells were transfected with MYC-Beclin 1 and 3 × FLAG tagged RhoB truncation mutants. Cell lysates were prepared at 48 h post transfection and subjected to western blotting analysis. The densities of MYC-Beclin 1 and LC3-II were normalized to that of β-Actin. The relative density of vector-transfected cells was set to 100%. n = 3 independent experiments. MYC-Beclin 1/β-Actin: Vector vs. WT P = 0.0085, Vector vs. 42-196 P = 0.9603, Vector vs. 89-196 P = 0.0197, Vector vs. 141-196 P = 0.9997, Vector vs. 1-180 P = 0.9998; LC3-II/β-Actin: Vector vs. WT P = 0.0019, Vector vs. 42-196 P = 0.9997, Vector vs. 89-196 P = 0.0404, Vector vs. 141-196 P = 0.9656, Vector vs. 1-180 P = 0.6522. **e**, Representative confocal images and statistic analysis of GFP-LC3 puncta in HEK 293 cells transfected with RhoB WT and truncation mutants. The number of GFP-LC3 puncta per cell in each group was calculated relative to that of vector-transfected cells (n = 10 random areas per group from three independent experiments), which was set to 100%. Scale bar, 10 μm. Vector vs. RhoB P < 1.0e-15, Vector vs. 42-196 P = 0.0832, Vector vs. 89-196 P < 1.0e-15, Vector vs. 118-196 P < 1.0e-15, Vector vs. 141-196 P = 0.7897, Vector vs. 1-180 P = 0.1677. **f**, Structure of RhoB (Protein Data Bank identifier 2FV8) with indicated binding sites for RhoB-Beclin 1 interaction. α-helices are shown in red with β-strands in yellow and loops in blue. **g**, Effect of RhoB single mutants at helical region on LC3 lipidation. The densities of MYC-Beclin 1 and LC3-II were normalized to that of β-Actin. The relative density of vector-transfected cells was set to 100%. n = 3 independent experiments. MYC-Beclin 1/β-Actin: Vector vs. RhoB P = 0.0071, Vector vs. T129V P = 0.2801, Vector vs. R133A P = 0.9998, Vector vs. Q136G P = 0.00406; LC3- II /β-Actin: Vector vs. RhoB P = 0.0360, Vector vs. T129V P = 0.0518, Vector vs. R133A P = 0.8942, Vector vs. Q136G P = 0.0282. **h**, Representative confocal images and statistic analysis of GFP-LC3 puncta in HEK 293 cells transfected with RhoB single mutants at helical region (n = 17 random areas per group from three independent experiments). The number of GFP-LC3 puncta per cell in vector-transfected cells was set to 100%. Scale bar, 10 μm. Vector vs. RhoB P < 1.0e-15, Vector vs. T129V P < 1.0e-15, Vector vs. R133A P = 0.4245, Vector vs. Q136G P < 1.0e-15. **i**, Bacterial survival in 5637 cells transfected with RhoB single mutant at helical region. n = 3 independent experiments. RhoB vs. Vector P < 1.0e-15, RhoB vs. T129V P > 0.9999, RhoB vs. R133A P = 0.0034, RhoB vs. Q136G P > 0.9999. **j**, Schematic diagram showing the interface of RhoB-Beclin 1 interaction. Data are the mean ± SD, *P < 0.05, **P < 0.01, ****P <0.0001, one-way ANOVA (b, d-e, g-i).

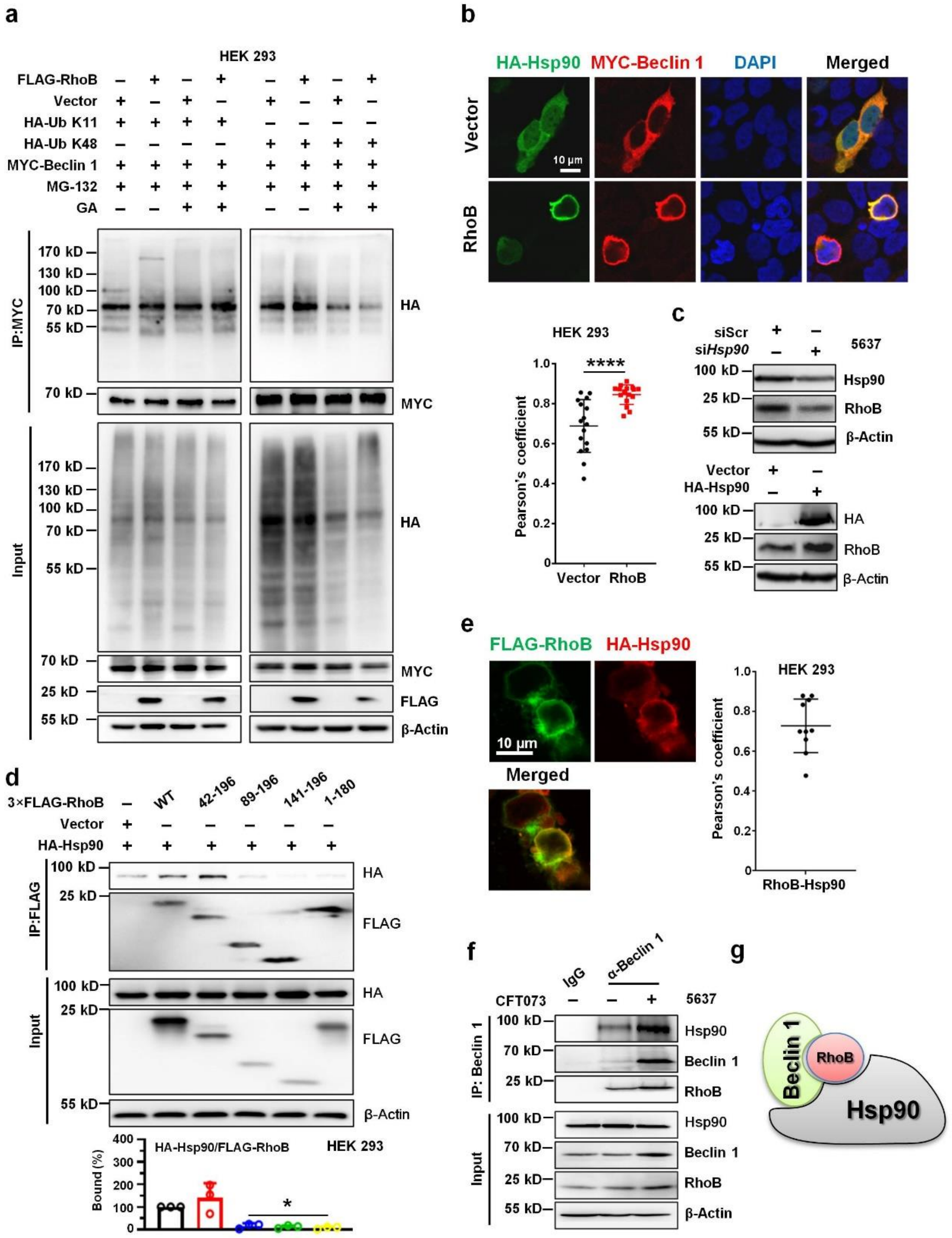

Supplementary Figure 6. RhoB is also a client protein of Hsp90 and forms a protein complex of RhoB-Beclin 1-Hsp90. a, Ubiquitination of Beclin 1 in *RhoB*-overexpressing cells with treatments of MG-132 and GA. HEK 293 cells were co-transfected with plasmids encoding MYC-Beclin 1 and HA-tagged ubiquitin (K11-linked Ub or K48-linked Ub), together with FLAG-RhoB or vector for 36 h. Transfected cells were treated with or without GA (1 μM) and MG-132 (10 μM) overnight. Cell lysates were then prepared and subjected to immunoprecipitation with anti-MYC antibody, followed by western blotting analysis. n = 3 independent

experiments. **b**, Representative confocal images of Beclin 1-Hsp90 colocalization in *RhoB*-overexpressing HEK 293 cells. HEK 293 cells were co-transfected with HA-Hsp90, MYC-Beclin 1 in the presence or absence of FLAG-RhoB (vector served as negative control) for 48 h, HA: green, Beclin 1: red. The Pearson correlation coefficients (*R* values) of Beclin 1-Hsp90 colocalization was calculated by Image Pro Plus software. n = 16 random areas from three independent experiments . Scale bar, 10 μm. Pearson's coefficient for Hsp90-Beclin 1 in vector- and *RhoB*-overexpressing cells were ~0.69 and ~0.85, respectively. P = 4.6e-5. **c**, Effect of Hsp90 on *RhoB* expression. 5637 cells were transfected with either *Hsp90* siRNAs (left) or HA-Hsp90 (right) for 48 h. n = 3 independent experiments. **d**, Interaction of Hsp90 with RhoB truncation mutants. HEK 293 cells were transfected with HA-Hsp90 and 3×FLAG *RhoB* truncation mutants for 48 h. The density of HA-Hsp90 was normalized to that of FLAG-RhoB in IP group. The percentage of bound HA-Hsp90 to RhoB WT was set to 100%. n = 3 independent experiments. HA-Hsp90/FLAG-RhoB: WT vs. 42-196 P = 0.3251, WT vs. 89-196 P = 0.0193, WT vs. 141-196 P = 0.0144, WT vs. 1-180 P = 0.0108. **e**, Colocalization of RhoB and Hsp90. HEK 293 cells were co-transfected with FLAG-RhoB and HA-Hsp90, followed by immunofluorescence staining. RhoB: green, HA: red. Representative images were shown (left). The Pearson correlation coefficients (*R* values) of RhoB-Hsp90 colocalization was calculated by Image Pro Plus software (right). n = 10 random areas from three independent experiments. Scale bar, 10 μm. Pearson's coefficient, ~0.73. **f**, The interaction of endogenous Hsp90 with Beclin 1 in 5637 cells infected with CFT073. At 2 hpi, cell lysates were prepared and subjected to immunoprecipitation assay with anti-Beclin 1 antibody or IgG, followed by western blotting analysis. n = 3 independent experiments. **g**, Schematic diagram of RhoB-Beclin1-Hsp90 complex. Data are the mean ± SD, *P <0.05, ****P < 0.0001, two-tailed unpaired Student's t test (b) or one-way ANOVA (d).

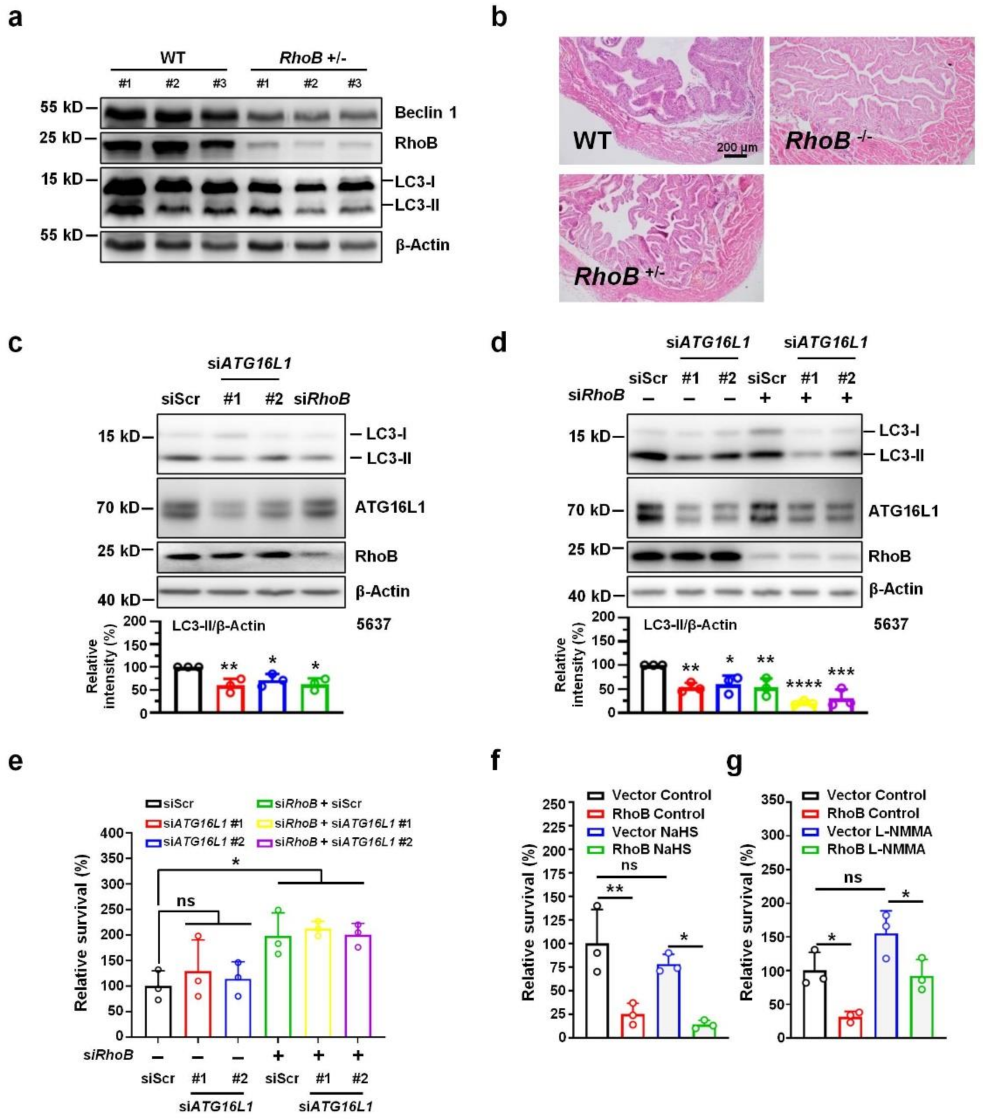

Supplementary Figure 7. *RhoB*$^{+/-}$ mice show decreased expression of autophagic proteins. a, Western blotting analysis of indicated proteins in CFT073-infected WT and *RhoB*$^{+/-}$ mice. n = 3 independent mice. **b**, Representative H&E staining of bladders of WT, *RhoB*$^{+/-}$ and *RhoB*$^{-/-}$ mice. Three independent experiments. Scale bar, 200 µm. **c-d,** Knockdown of *ATG16L1* has no effect on RhoB level in 5637 bladder epithelial cells with **(d)** or without **(c)** CFT073 infection. n = 3 independent experiments. (**c**) LC3-II/β-Actin: siScr vs. si*ATG16L1* #1 *P* = 0.0079, siScr vs. si*ATG16L1* #2 *P* = 0.0453, siScr vs. si*RhoB* *P* = 0.0126. (**d**) LC3-II/β-Actin: si*Scr* vs. si*ATG16L1* #1 *P* = 0.0061, siScr vs. si*ATG16L1* #2 *P* = 0.0178, siScr vs. si*RhoB* *P* = 0.0064, siScr vs. si*RhoB* plus si*ATG16L1* #1 *P* <1.0e-15, siScr vs. si*RhoB* plus si*ATG16L1* #2 *P* = 0.0003. **e**, Knockdown of *ATG16L1* has no significant effect on RhoB-mediated CFT073 clearance in 5637 bladder epithelial cells. n = 3 independent experiments. siScr vs. si*ATG16L1* #1 *P* = 0.8013, siScr vs. si*ATG16L1* #2 *P* = 0.9852, siScr vs. si*RhoB* *P* = 0.0293, siScr vs. si*RhoB* plus si*ATG16L1* #1 *P* = 0.0131, siScr vs. si*RhoB* plus si*ATG16L1* #2 *P* = 0.0261. **f-g,** Bacterial survival of CFT073 in *RhoB*-overexpressing

5637 cells treated with inhibitors, 1 mM NaHS **(f)** or 10 mM L-NMMA **(g)**. Inhibitors were added to the culture medium at 30 min before infection and maintained during experiments. n = 3 independent experiments. (**f**) Vector Control vs. RhoB Control P = 0.0042, Vector Control vs. Vector NaHS P = 0.4407, Vector NaHS vs. RhoB NaHS P = 0.0102. (**g**) Vector Control vs. RhoB Control P = 0.0238, Vector Control vs. Vector L-NMMA P = 0.0641, Vector L-NMMA vs. RhoB L-NMMA P = 0.0358. Data are the mean ± SD, *P < 0.05, **P < 0.01, ***P < 0.001, ****P < 0.0001; ns, not significant; one-way ANOVA (c-g).

Supplementary Figure 8

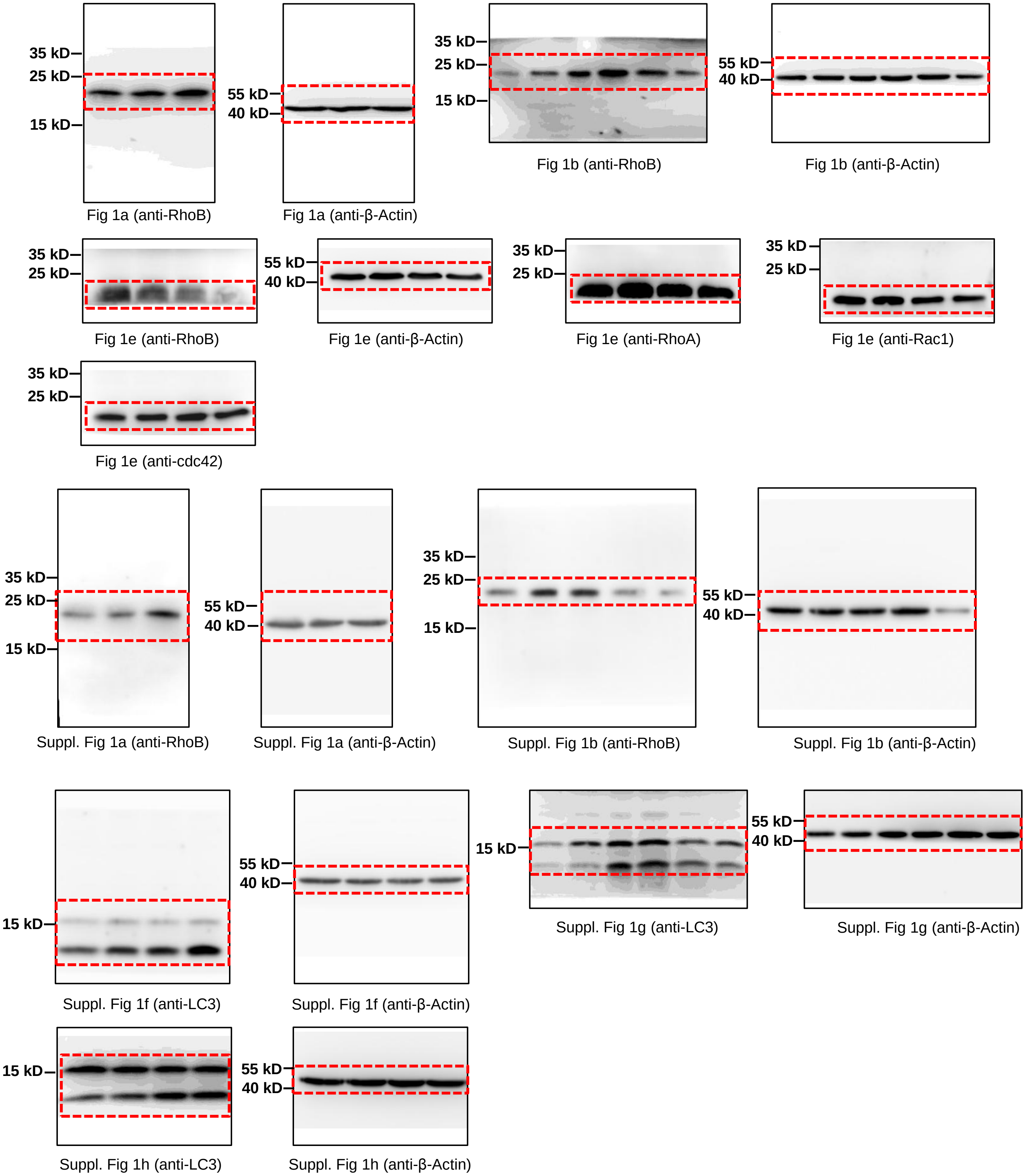

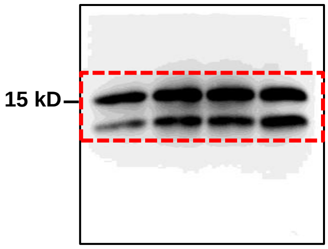

Fig 2a (anti-LC3)

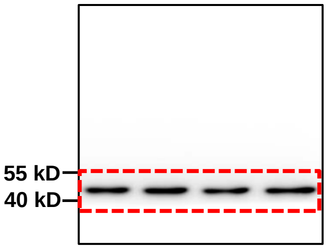

Fig 2a (anti-β-Actin)

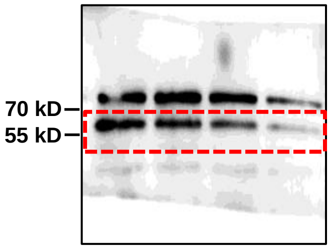

Fig 2a (anti-P62)

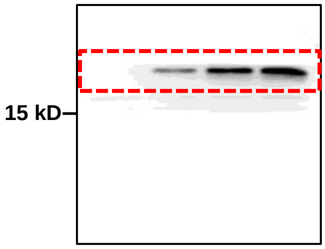

Fig 2a (anti-FLAG)

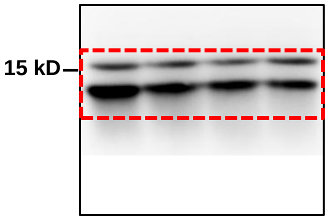

Fig 2d (anti-LC3)

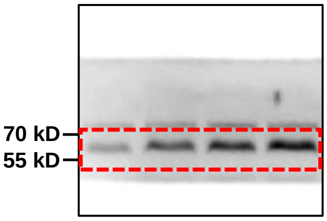

Fig 2d (anti-P62)

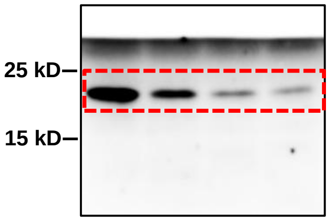

Fig 2d (anti-RhoB)

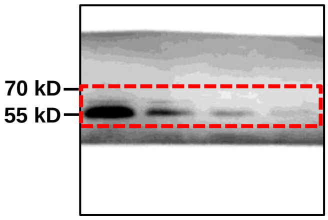

Fig 2d (anti-Beclin 1)

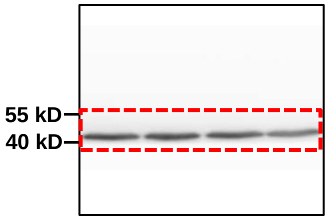

Fig 2d (anti-β-Actin)

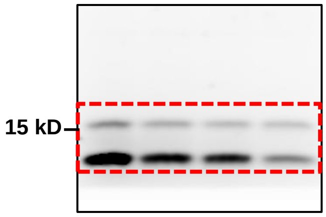

Fig 2e (anti-LC3)

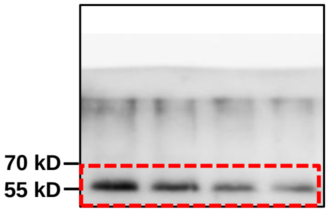

Fig 2e (anti-Beclin 1)

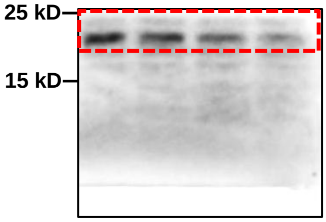

Fig 2e (anti-RhoB)

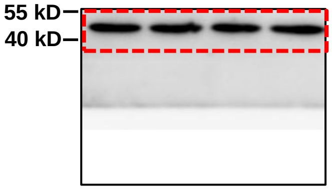

Fig 2e (anti-β-Actin)

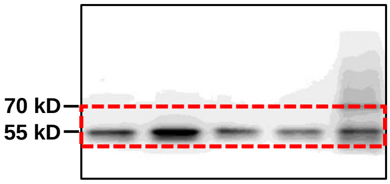

Fig 2f (anti-Beclin 1)

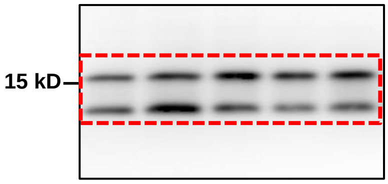

Fig 2f (anti-LC3)

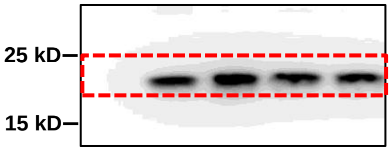

Fig 2f (anti-FLAG)

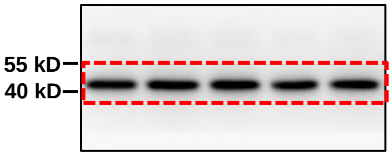

Fig 2f (anti-β-Actin)

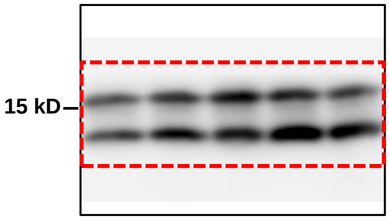

Fig 2h (anti-LC3)

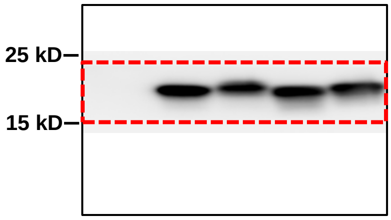

Fig 2h (anti-FLAG)

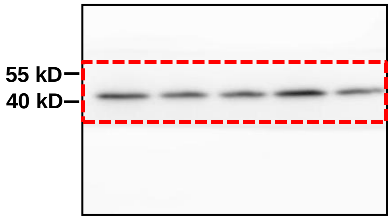

Fig 2h (anti-β-Actin)

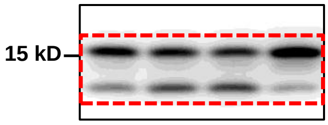

Fig 2i (anti-LC3)

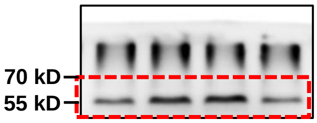

Fig 2i (anti-Beclin 1)

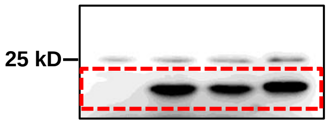

Fig 2i (anti-FLAG)

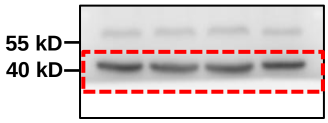

Fig 2i (anti-β-Actin)

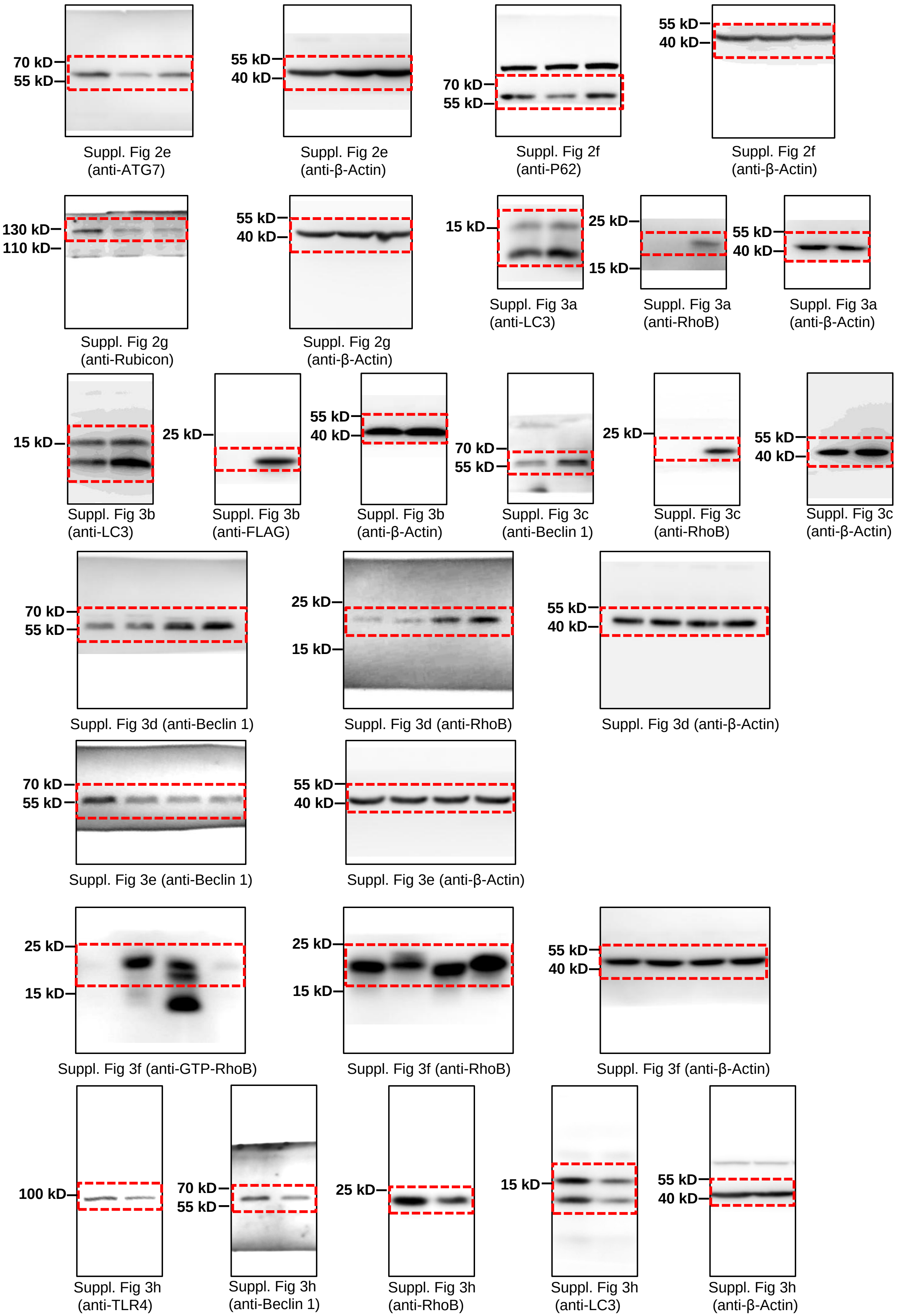
70 kD
55 kD
Suppl. Fig 2e (anti-ATG7)
55 kD
40 kD
Suppl. Fig 2e (anti-β-Actin)
70 kD
55 kD
Suppl. Fig 2f (anti-P62)
55 kD
40 kD
Suppl. Fig 2f (anti-β-Actin)
130 kD
110 kD
Suppl. Fig 2g (anti-Rubicon)
55 kD
40 kD
Suppl. Fig 2g (anti-β-Actin)
15 kD
Suppl. Fig 3a (anti-LC3)
25 kD
15 kD
Suppl. Fig 3a (anti-RhoB)
55 kD
40 kD
Suppl. Fig 3a (anti-β-Actin)
15 kD
Suppl. Fig 3b (anti-LC3)
25 kD
Suppl. Fig 3b (anti-FLAG)
55 kD
40 kD
Suppl. Fig 3b (anti-β-Actin)
70 kD
55 kD
Suppl. Fig 3c (anti-Beclin 1)
25 kD
Suppl. Fig 3c (anti-RhoB)
55 kD
40 kD
Suppl. Fig 3c (anti-β-Actin)
70 kD
55 kD
Suppl. Fig 3d (anti-Beclin 1)
25 kD
15 kD
Suppl. Fig 3d (anti-RhoB)
55 kD
40 kD
Suppl. Fig 3d (anti-β-Actin)
70 kD
55 kD
Suppl. Fig 3e (anti-Beclin 1)
55 kD
40 kD
Suppl. Fig 3e (anti-β-Actin)
25 kD
15 kD
Suppl. Fig 3f (anti-GTP-RhoB)
25 kD
15 kD
Suppl. Fig 3f (anti-RhoB)
55 kD
40 kD
Suppl. Fig 3f (anti-β-Actin)
100 kD
Suppl. Fig 3h (anti-TLR4)
70 kD
55 kD
Suppl. Fig 3h (anti-Beclin 1)
25 kD
Suppl. Fig 3h (anti-RhoB)
15 kD
Suppl. Fig 3h (anti-LC3)
55 kD
40 kD
Suppl. Fig 3h (anti-β-Actin)

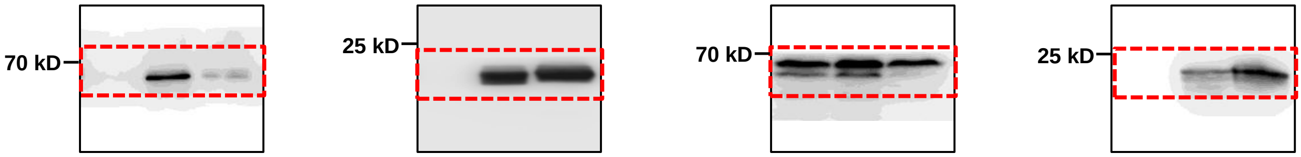

Fig 3a IP(anti-MYC) Fig 3a IP (anti-FLAG) Fig 3a input (anti-MYC) Fig 3a input (anti-FLAG)

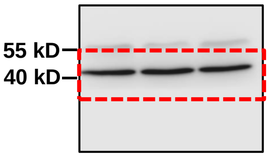

Fig 3a input (anti-β-Actin)

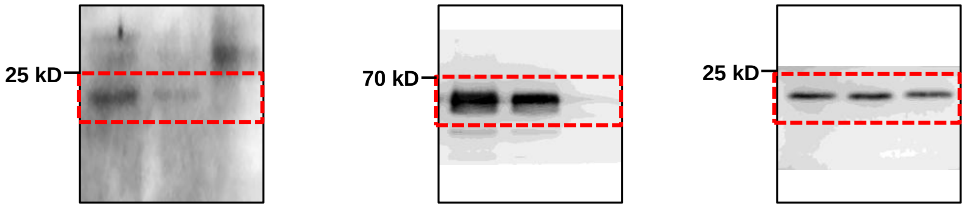

Fig 3b IP (anti-FLAG) Fig 3b IP (anti-MYC) Fig 3b input (anti-FLAG)

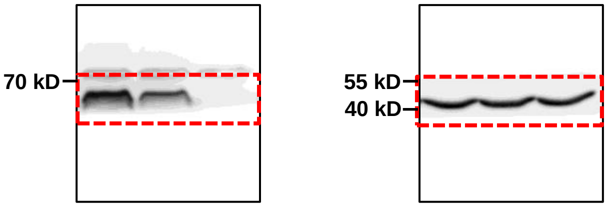

Fig 3b input (anti-MYC) Fig 3b input (anti-β-Actin)

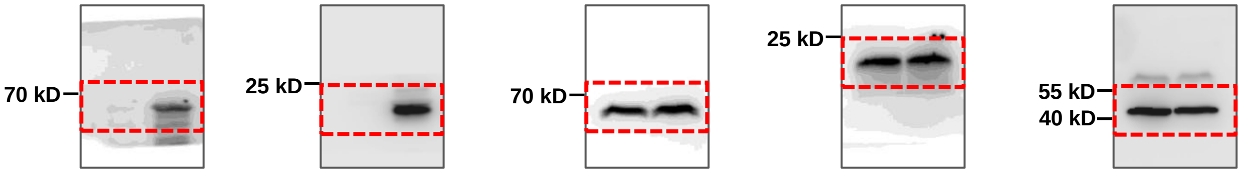

Fig 3c IP(anti-Beclin 1) Fig 3c IP (anti-RhoB) Fig 3c input (anti-Beclin 1) Fig 3c input (anti-RhoB) Fig 3c input (anti-β-Actin)

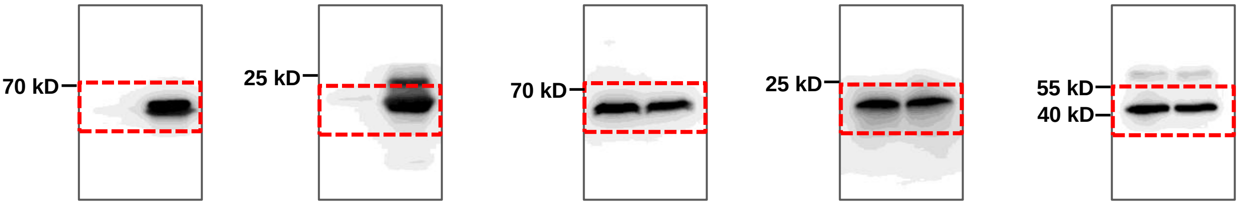

Fig 3d IP (anti-Beclin 1) Fig 3d IP (anti-RhoB) Fig 3d input (anti-Beclin 1) Fig 3d input (anti-RhoB) Fig 3d input (anti-β-Actin)

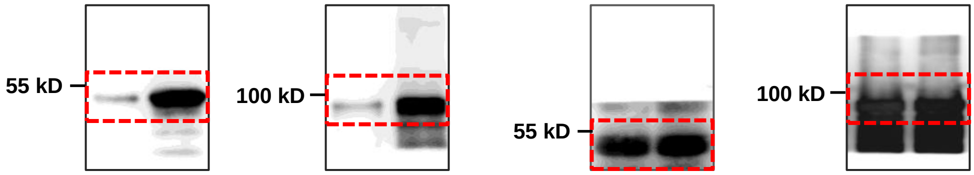

Fig 3e IP (anti-RhoB) Fig 3e IP(anti-Beclin 1) Fig 3e input (anti-RhoB) Fig 3e input (anti-Beclin 1)

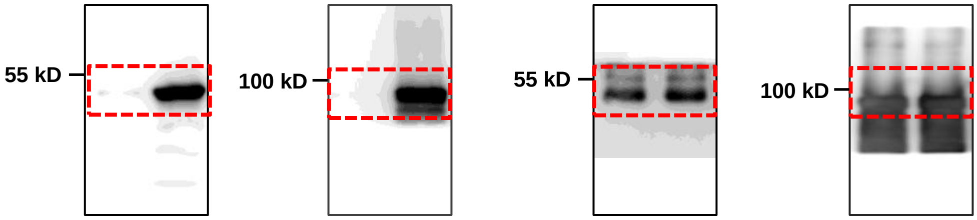

Fig 3f IP (anti-RhoB) Fig 3f IP(anti-Beclin 1) Fig 3f input (anti-RhoB) Fig 3f input (anti-Beclin 1)

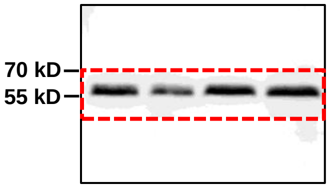

Suppl. Fig 4b (anti-MYC)

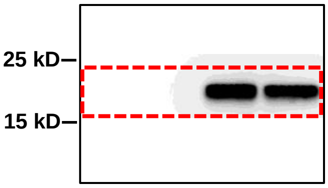

Suppl. Fig 4b (anti-FLAG)

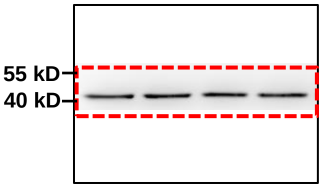

Suppl. Fig 4b (anti-β-Actin)

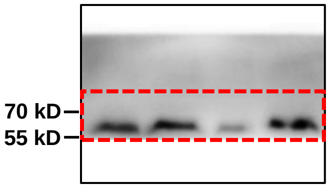

Suppl. Fig 4c (anti-Beclin 1)

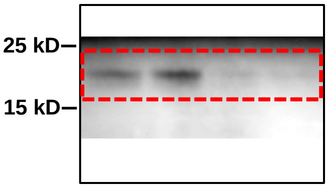

Suppl. Fig 4c (anti-RhoB)

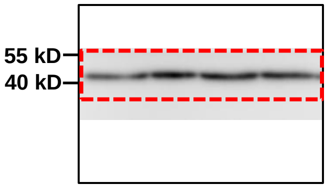

Suppl. Fig 4c (anti-β-Actin)

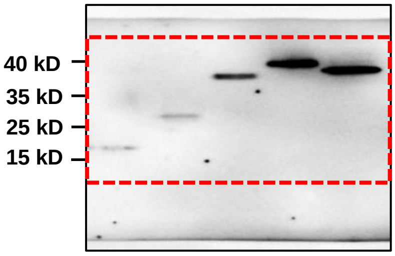

Fig 4a IP(anti-MYC)

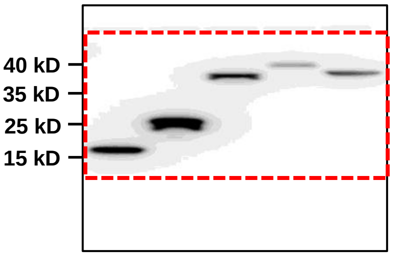

Fig 4a input (anti-MYC)

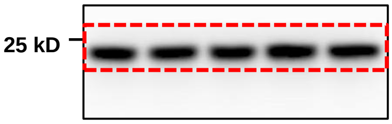

Fig 4a IP (anti-FLAG)

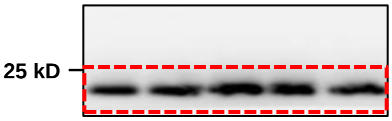

Fig 4a input (anti-FLAG)

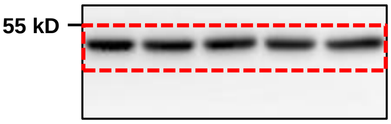

Fig 4a input (anti-β-Actin)

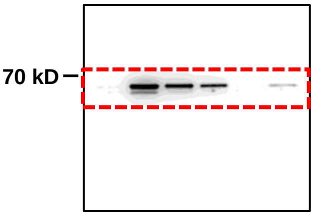

Fig 4b IP (anti-MYC)

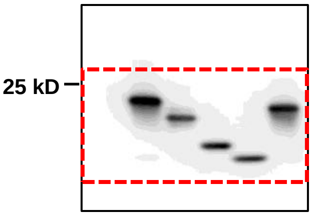

Fig 4b IP (anti-FLAG)

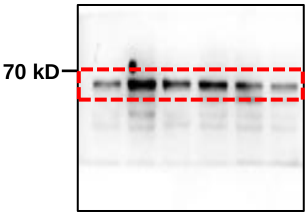

Fig 4b input (anti-MYC)

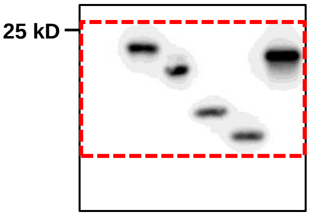

Fig 4b input (anti-FLAG)

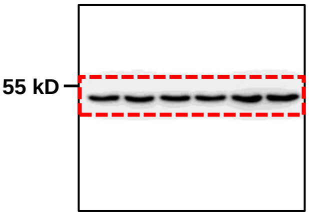

Fig 4b input (anti-β-Actin)

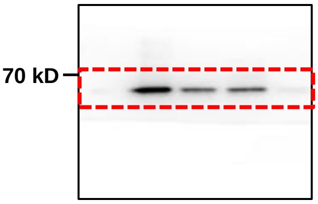

Fig 4c IP (anti-MYC)

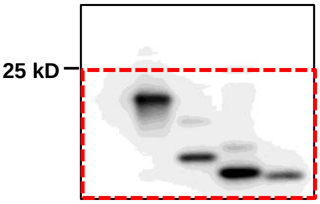

Fig 4c IP (anti-FLAG)

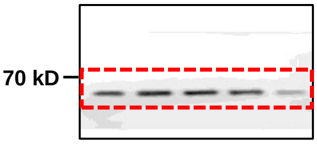

Fig 4c input (anti-MYC)

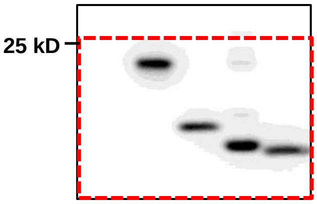

Fig 4c input (anti-FLAG)

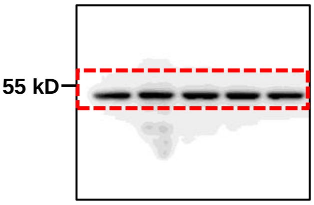

Fig 4c input (anti-β-Actin)

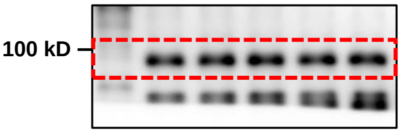

Fig 4d IP (anti-Beclin 1)

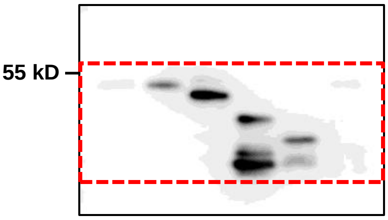

Fig 4d IP (anti-GST-RhoB)

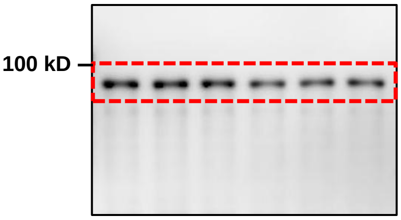

Fig 4d input (anti-Beclin 1)

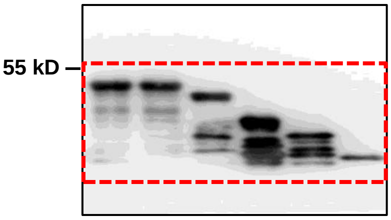

Fig 4d input (anti-GST-RhoB)

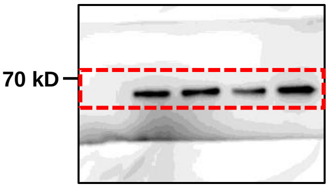

Fig 4e IP (anti-MYC)

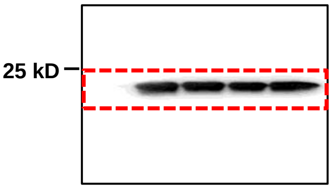

Fig 4e IP (anti-FLAG)

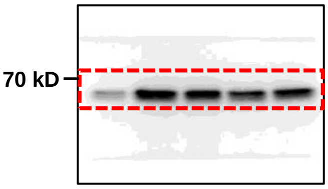

Fig 4e input (anti-MYC)

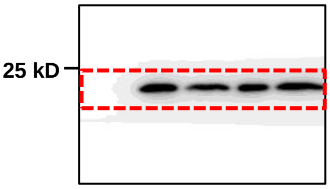

Fig 4e input (anti-FLAG)

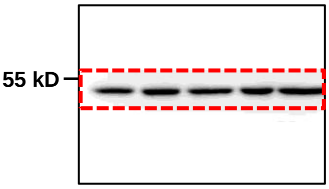

Fig 4e input (anti-β-Actin)

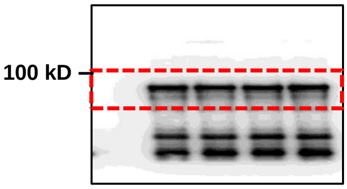

Fig 4f IP (anti-Beclin 1)

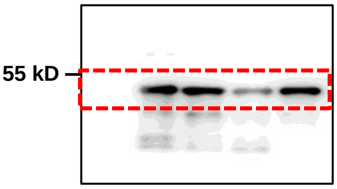

Fig 4f IP (anti-GST-RhoB)

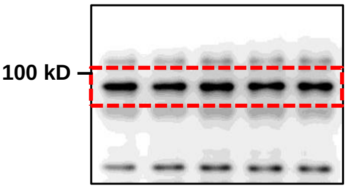

Fig 4f input (anti-Beclin 1)

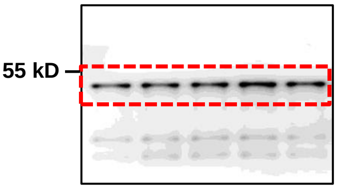

Fig 4f input (anti-GST-RhoB)

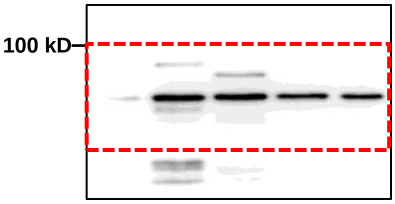

Suppl. Fig 5b IP (anti-GST)

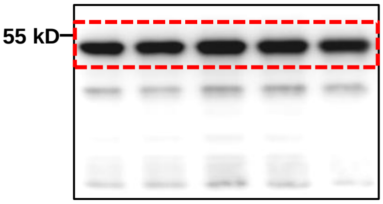

Suppl. Fig 5b input (anti-GST-RhoB)

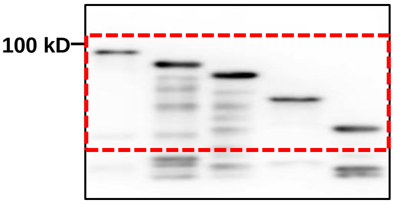

Suppl. Fig 5b input (anti-GST-Beclin 1)

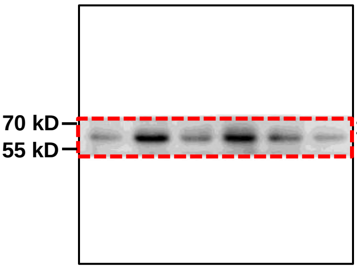

Suppl. Fig 5d (anti-MYC)

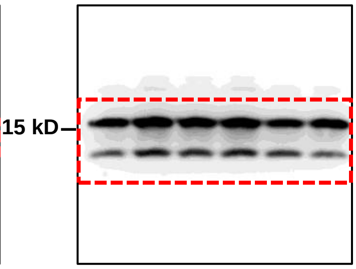

Suppl. Fig 5d (anti-LC3)

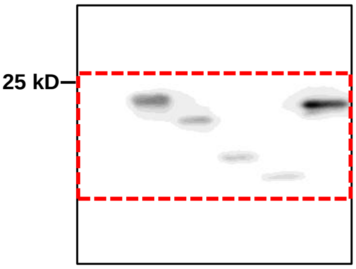

Suppl. Fig 5d (anti-FLAG)

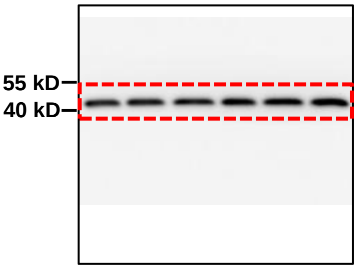

Suppl. Fig 5d (anti-β-Actin)

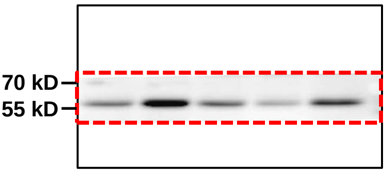

Suppl. Fig 5g (anti-MYC)

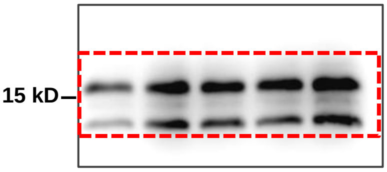

Suppl. Fig 5g (anti-LC3)

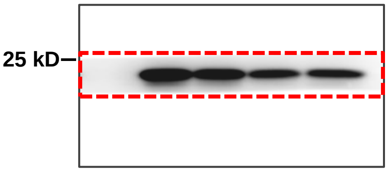

Suppl. Fig 5g (anti-FLAG)

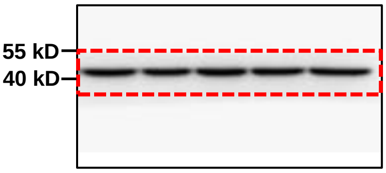

Suppl. Fig 5g (anti-β-Actin)

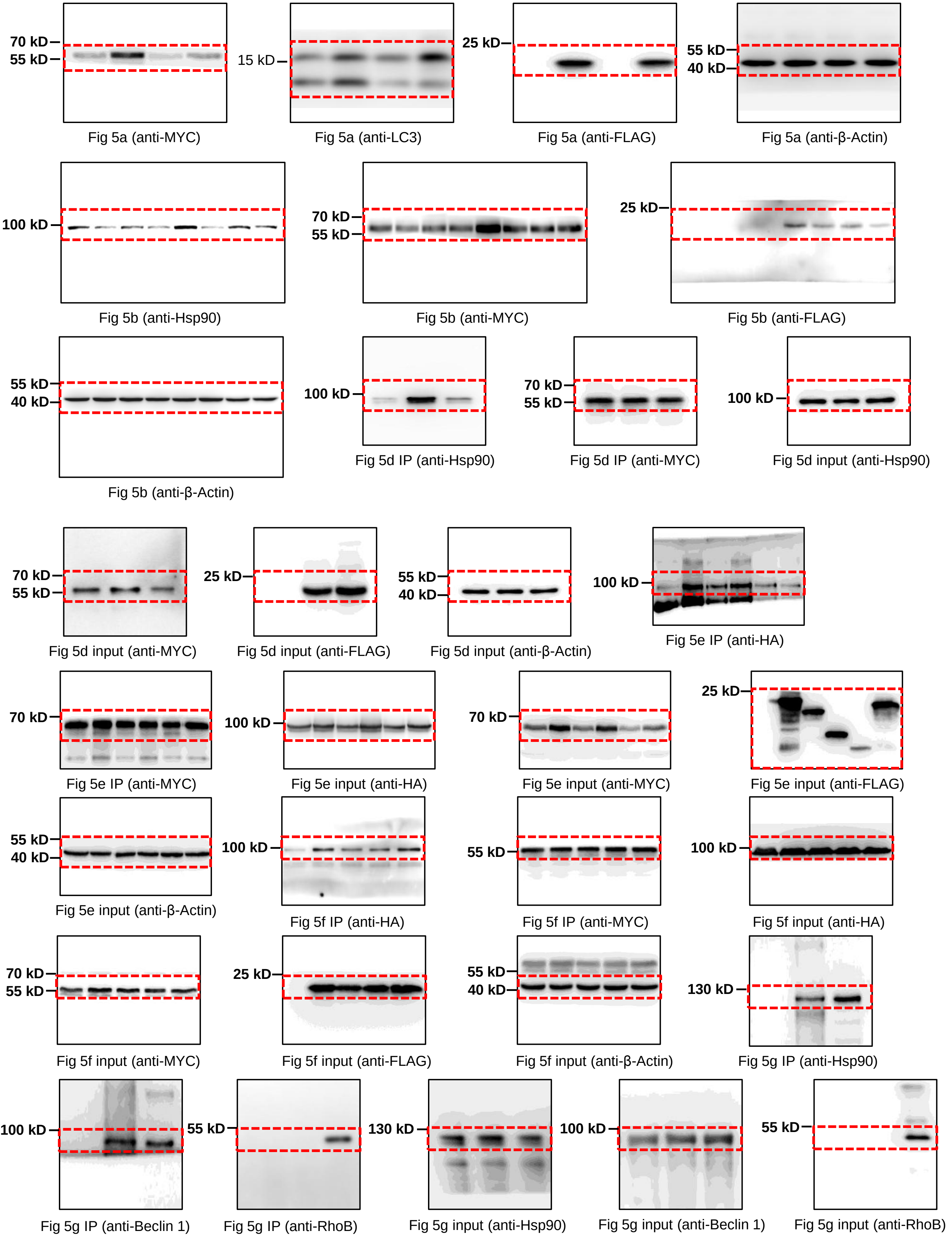

70 kD
55 kD
Fig 5a (anti-MYC)
15 kD
Fig 5a (anti-LC3)
25 kD
Fig 5a (anti-FLAG)
55 kD
40 kD
Fig 5a (anti-β-Actin)
100 kD
Fig 5b (anti-Hsp90)
70 kD
55 kD
Fig 5b (anti-MYC)
25 kD
Fig 5b (anti-FLAG)
55 kD
40 kD
Fig 5b (anti-β-Actin)
100 kD
Fig 5d IP (anti-Hsp90)
70 kD
55 kD
Fig 5d IP (anti-MYC)
100 kD
Fig 5d input (anti-Hsp90)
70 kD
55 kD
Fig 5d input (anti-MYC)
25 kD
Fig 5d input (anti-FLAG)
55 kD
40 kD
Fig 5d input (anti-β-Actin)
100 kD
Fig 5e IP (anti-HA)
70 kD
Fig 5e IP (anti-MYC)
100 kD
Fig 5e input (anti-HA)
70 kD
Fig 5e input (anti-MYC)
25 kD
Fig 5e input (anti-FLAG)
55 kD
40 kD
Fig 5e input (anti-β-Actin)
100 kD
Fig 5f IP (anti-HA)
55 kD
Fig 5f IP (anti-MYC)
100 kD
Fig 5f input (anti-HA)
70 kD
55 kD
Fig 5f input (anti-MYC)
25 kD
Fig 5f input (anti-FLAG)
55 kD
40 kD
Fig 5f input (anti-β-Actin)
130 kD
Fig 5g IP (anti-Hsp90)
100 kD
Fig 5g IP (anti-Beclin 1)
55 kD
Fig 5g IP (anti-RhoB)
130 kD
Fig 5g input (anti-Hsp90)
100 kD
Fig 5g input (anti-Beclin 1)
55 kD
Fig 5g input (anti-RhoB)

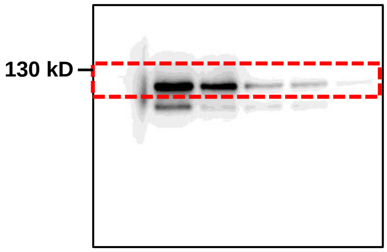

Fig 5h IP (anti-Hsp90)

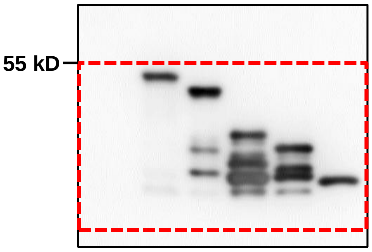

Fig 5h IP (anti-RhoB)

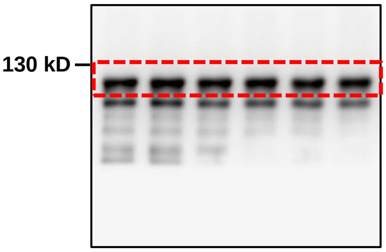

Fig 5h input (anti-Hsp90)

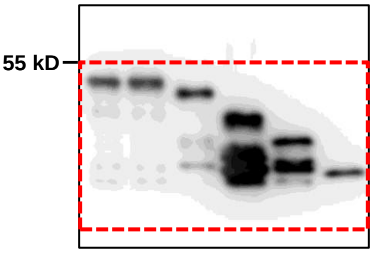

Fig 5h input (anti-RhoB)

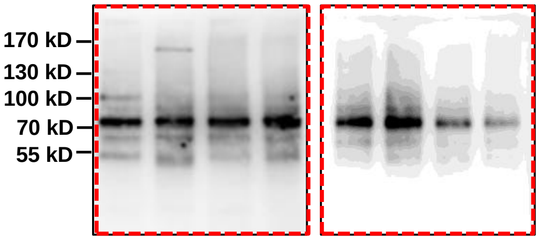

Suppl. Fig 6a IP (anti-HA)

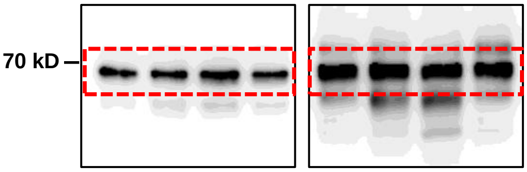

Suppl. Fig 6a IP (anti-MYC)

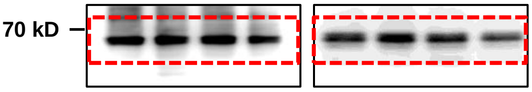

Suppl. Fig 6a Iinput (anti-MYC)

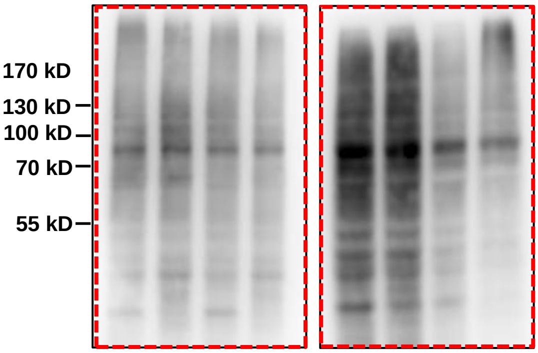

Suppl. Fig 6a input (anti-HA)

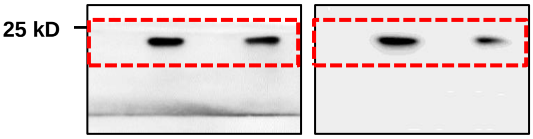

Suppl. Fig 6a input (anti-FLAG)

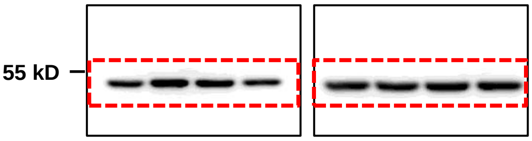

Suppl. Fig 6a input (anti-β-Actin)

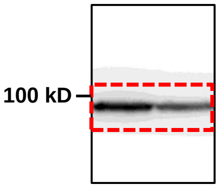

Suppl. Fig 6c-top
(anti-Hsp90)

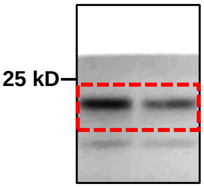

Suppl. Fig 6c-top
(anti-RhoB)

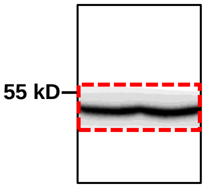

Suppl. Fig 6c-top
(anti- β-Actin)

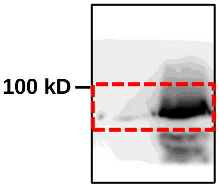

Suppl. Fig 6c-bottom
(anti-HA)

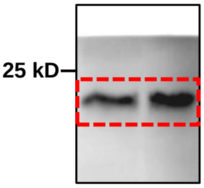

Suppl. Fig 6c-bottom
(anti-RhoB)

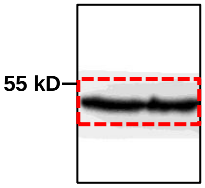

Suppl. Fig 6c-bottom
(anti- β-Actin)

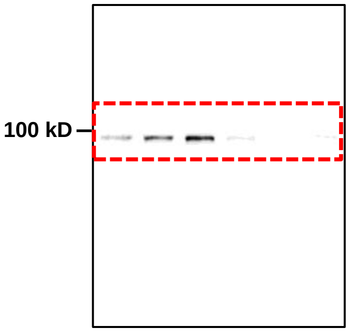

Suppl. Fig 6d IP (anti-HA)

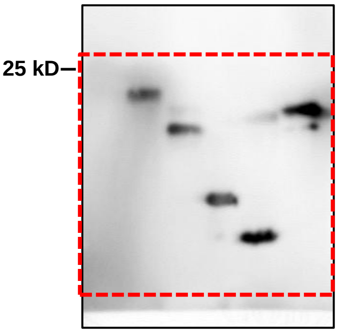

Suppl. Fig 6d IP (anti-FLAG)

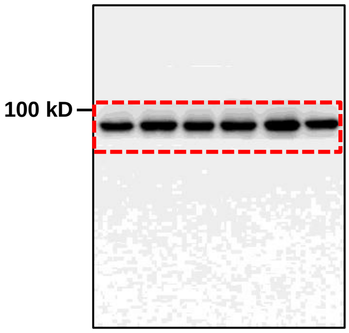

Suppl. Fig 6d input (anti-HA)

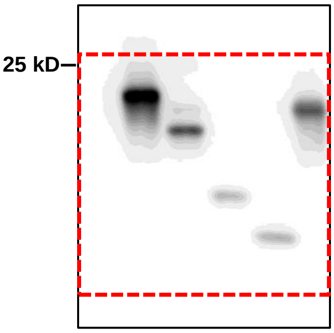

Suppl. Fig 6d input (anti-FLAG)

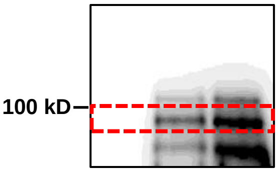

Suppl. Fig 6f IP (anti-Hsp90)

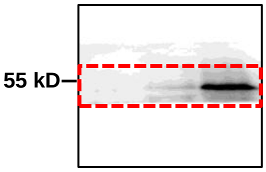

Suppl. Fig 6f IP (anti-Beclin 1)

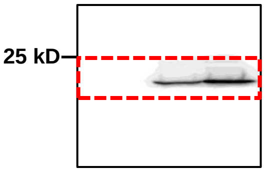

Suppl. Fig 6f IP (anti-RhoB)

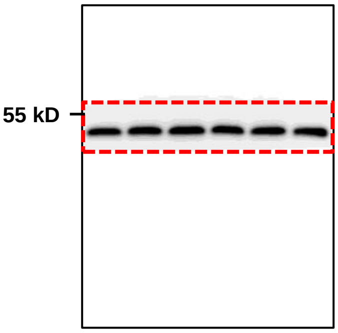

Suppl. Fig 6d input (anti- β-Actin)

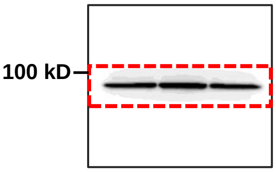

Suppl. Fig 6f input (anti-Hsp90)

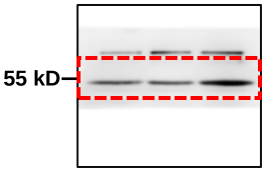

Suppl. Fig 6f input (anti-Beclin 1)

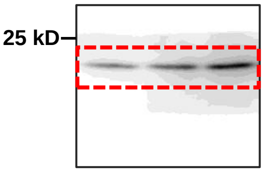

Suppl. Fig 6f input (anti-RhoB)

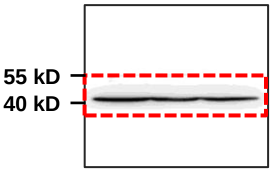

Suppl. Fig 6f (anti-β-Actin)

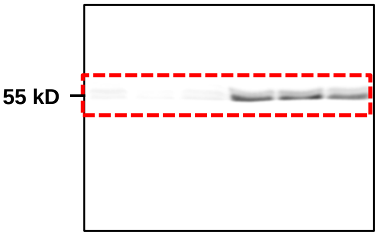

Fig 6b (anti-Beclin 1)

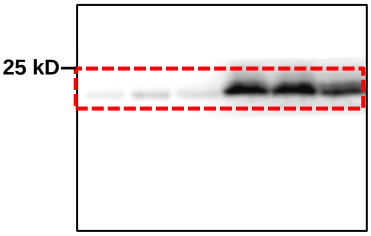

Fig 6b (anti-RhoB)

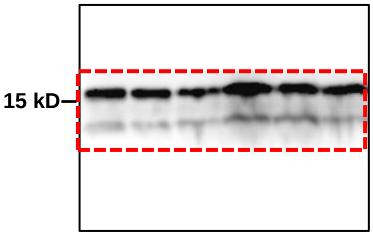

Fig 6b (anti-LC3)

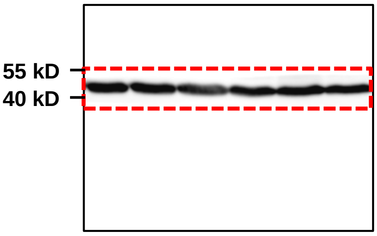

Fig 6b (anti-β-Actin)

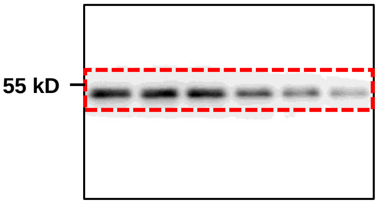

Fig 6d (anti-Beclin 1)

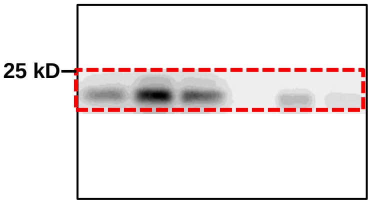

Fig 6d (anti-RhoB)

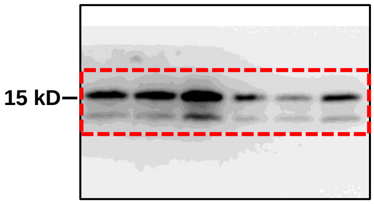

Fig 6d (anti-LC3)

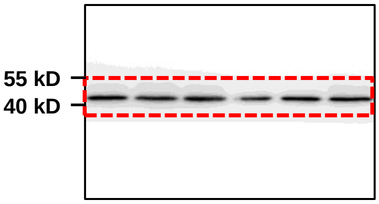

Fig 6d (anti-β-Actin)

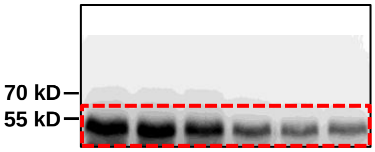

Suppl. Fig 7a (anti-Beclin 1)

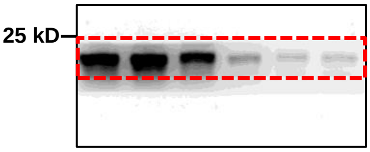

Suppl. Fig 7a (anti-RhoB)

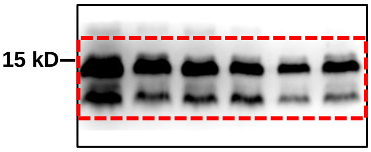

Suppl. Fig 7a (anti-LC3)

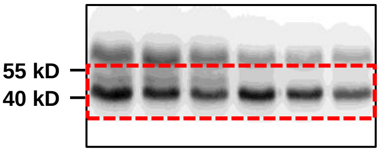

Suppl. Fig 7a (anti-β-Actin)

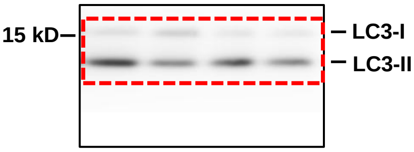

Suppl. Fig 7c (anti-LC3)

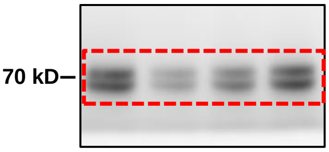

Suppl. Fig 7c (anti-ATG16L1)

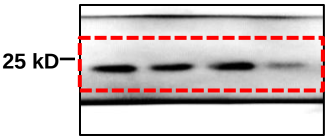

Suppl. Fig 7c (anti-RhoB)

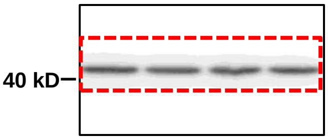

Suppl. Fig 7c (anti-β-Actin)

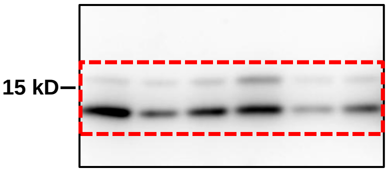

Suppl. Fig 7d (anti-LC3)

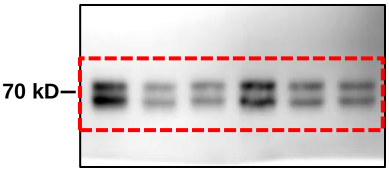

Suppl. Fig 7d (anti-ATG16L1)

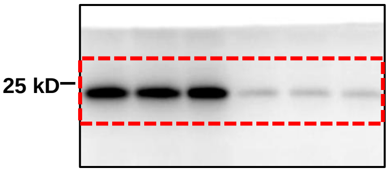

Suppl. Fig 7d (anti-RhoB)

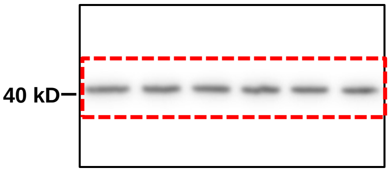

Suppl. Fig 7d (anti-β-Actin)
