# Supplementary material for: An infection-induced RhoB-Beclin 1-Hsp90 complex enhances clearance of uropathogenic Escherichia coli
Source: Nat Commun. 2021 May 10;12:2587. doi: 10.1038/s41467-021-22726-8 (PMC8110956; doi:10.1038/s41467-021-22726-8)
Supplement: Supplementary file 4 — Description of Additional Supplementary Files [file 41467_2021_22726_MOESM4_ESM.docx]

Description of additional supplementary information

Title: Supplementary Data 1

Description: Strains and plasmids in this study.

Title: Supplementary Data 2

Description: Primers and siRNAs in this study.
